# Clinical and Psychological Outcomes After Monoclonal Gammopathy Screening: A Population-Based Screening Study and Subsequent Randomized Trial of Follow-Up

**DOI:** 10.1200/JCO-25-02771

**Published:** 2026-07-14

**Authors:** Sæmundur Rögnvaldsson, Sigrún Thorsteinsdóttir, Elias Eythorsson, Inga Dröfn Wessman, Gudrun Asta Sigurdardottir, Brynjar Vidarsson, Pall T. Onundarson, Bjarni Agnarsson, Margret Sigurdardottir, Isleifur Olafsson, Ingunn Thorsteinsdottir, Andri Steinthor Bjornsson, Jon Thorir Oskarsson, Andri Olafsson, Gauti Gislason, Ingigerdur Sverrisdottir, Thorir E. Long, Elfa Rún Guðmundsdóttir, Asdis Rosa Thordardottir, Asbjorn Jonsson, Runolfur Palsson, Olafur S. Indridason, Malin Hultcrantz, Brian G.M. Durie, Stephen J. Harding, Thor Aspelund, Ola Landgren, Thorvardur Jon Love, Sigurdur Y. Kristinsson

**Affiliations:** ^1^ Faculty of Medicine, University of Iceland, Reykjavik, Iceland; ^2^ Sahlgrenska University Hospital, Gothenburg, Sweden; ^3^ Institute of Medicine, Sahlgrenska Academy, University of Gothenburg, Gothenburg, Sweden; ^4^ Rigshospitalet, Copenhagen, Denmark; ^5^ Landspitali University Hospital, Reykjavik, Iceland; ^6^ Faculty of Psychology, University of Iceland, Reykjavik, Iceland; ^7^ Skane University Hospital, Lund, Sweden; ^8^ Akureyri Hospital, Akureyri, Iceland; ^9^ Myeloma Service, Department of Medicine, Memorial Sloan Kettering Cancer Center, New York, NY; ^10^ Samuel Oschin Comprehensive Cancer Institute, Cedars-Sinai Outpatient Cancer Center, Los Angeles, CA; ^11^ Binding Site Group Ltd Part of Thermo Fisher Scientific, Birmingham, United Kingdom; ^12^ Division of Myeloma Program, Department of Medicine, University of Miami, Sylvester Comprehensive Cancer Center, Miami, FL

## Abstract

Multiple myeloma (MM) and related malignancies develop from an asymptomatic precursor condition, monoclonal gammopathy of undetermined significance (MGUS), detectable via blood testing. The benefits and harms of population-based MGUS screening remain uncertain. We conducted a nationwide screening study for monoclonal gammopathies and a randomized trial of follow-up strategies in Iceland (Iceland Screens, Treats, or Prevents Multiple Myeloma; ClinicalTrials.gov identifier: NCT03327597). In total, 75,422 (53% of those invited) were screened and those with MGUS (n = 3,541) were randomly assigned to no notification (arm 1), guideline-based follow-up (arm 2), or intensified follow-up (arm 3). Progression, symptom burden, and psychological well-being were compared between the control arm (arm 1) and intervention arms (arms 2 and 3). After a median follow-up of 4.5 years, screening led to a 27-fold increase in the detection of smoldering MM (8.6% *v* 0.3%; hazard ratio, 27.46 [95% CI, 10.21 to 73.86]; *P* < .001), but active malignancy rates did not differ. Active MM and related malignancy were diagnosed 1 year earlier in the intervention arms with fewer symptomatic presentations and hospitalizations at diagnosis. MGUS notification was not associated with adverse psychological outcomes. These findings demonstrate that population-based screening facilitates earlier detection of MM and expands access to early intervention without detectable psychological harm. Longer follow-up is required to determine the effects on survival and cost-effectiveness.

## INTRODUCTION

Multiple myeloma (MM) and other symptomatic monoclonal gammopathies are preceded by monoclonal gammopathy of undetermined significance (MGUS),^1-3^ an asymptomatic disorder with an annual progression risk to active malignancy of 0.5%-1%.^4,5^ Follow-up is recommended for MGUS to detect progression before end-organ damage. This is particularly important in MM, where disease burden at diagnosis is associated with morbidity and mortality^6,7^ and prior diagnosis of MGUS has been associated with lower disease burden and improved survival.^8-10^ Initiating treatment at the intermediate precursor stage of high-risk smoldering MM (SMM) can delay progression to active MM and improve survival.^11-13^ However, similar to MGUS, SMM is asymptomatic and thus rarely encountered in clinical practice and 93%-97% of patients with MM are diagnosed at a symptomatic stage when it is too late for early treatment.^8-10^ Screening may present a way to detect these precursors and extend the impact of early treatment to the wider MM patient population.

Screening is one of the cornerstones of oncology but is controversial and can carry significant harm,^14^ including overdiagnosis and overtreatment, health care resource use and financial toxicity, and psychological distress. Despite the widespread implementation of cancer screening programs, the psychological effects of screening remain poorly studied.^15^

The Iceland Screens, Treats, or Prevents Multiple Myeloma (iStopMM) study is a population-based MGUS screening study and a randomized clinical trial of follow-up strategies. To our knowledge, it is the first clinical study designed to determine the benefits and harms of population-based screening for monoclonal gammopathies. The primary outcome of the study is overall survival, which remains immature. Here, we report secondary outcomes on early clinical and psychological effects of screening.

## METHODS

All residents of Iceland, born in 1975 or before and alive in September 2016 (148,711), were invited to participate; 80,759 (54%) provided their informed consent, of whom 75,422 (93%) were screened for monoclonal gammopathy. Serum samples were collected between 2016 and 2020 in parallel with other blood testing ordered within the universal Icelandic health care system, supplemented by a targeted blood sample collection drive, with relatively stable accrual across the screening period (Data Supplement, Fig S1, online only). Samples were screened by serum protein electrophoresis (Helena Laboratories, Beaumont, TX) and free light chain (FLC) assay (Freelite, The Binding Site, part of Thermo Fisher Scientific, Birmingham, UK), with immunofixation electrophoresis performed in cases of suspected M proteins or abnormal FLC testing. Those with newly detected apparent MGUS were randomly assigned to three study arms using block random assignment by M protein concentrations ≥1.5 g/dL and light-chain MGUS (Data Supplement, Table S1).^16,17^ Arm 1 was the control arm where participants were not notified of the MGUS status and continued as if they had not been screened. Arm 2 and arm 3 were the intervention arms that were notified and offered clinical assessment and follow-up either using a risk-adapted use of imaging and bone marrow sampling according to guidelines^18^ (arm 2) or a uniform intensive approach with bone marrow sampling for all (arm 3; Data Supplement, Table S2). Those who developed intermediate- or high-risk SMM were offered early treatment with lenalidomide and dexamethasone, or carfilzomib, lenalidomide, and dexamethasone, respectively, in a single-arm clinical study (ClinicalTrials.gov identifier: NCT03815279). Detailed methods are provided in the Methods Supplement and have been described previously.^19^

## RESULTS

Participant flow and characteristics are shown in the Data Supplement (Fig S2) and Table 1, respectively. After a median follow-up of 4.5 years, the intervention arms were 27.5-fold more likely to be diagnosed with SMM (8.6% *v* 0.3%; hazard ratio [HR], 27.46 [95% CI, 10.21 to 73.86]; *P* < .001; Data Supplement, Table S3 and Fig S3). Participants who were randomly assigned to intensified follow-up (arm 3) were more likely to be diagnosed with SMM than those receiving guideline-directed follow-up (arm 2; HR, 1.65 [95% CI, 1.24 to 2.19]; *P* < .001; Data Supplement, Table S3, Fig 1), driven by a higher SMM rate in low-risk MGUS (Data Supplement, Table S4). Most cases of SMM were low- or intermediate-risk SMM (94%, Data Supplement, Table S5). The incidence of active MM, the composite end point of MM and related malignancies, or symptomatic myeloma did not differ (HR, 0.83 [95% CI, 0.44 to 1.54]; *P* = .51, HR, 0.88 [95% CI, 0.59 to 1.14]; *P* = .55, and HR, 0.62 [95% CI, 0.31 to 1.21]; *P* = .16, respectively; Data Supplement, Figs S3 and S4). Participants in the intervention arms age 40-70 years at screening had a lower risk of active MM (HR, 0.56 [95% CI, 0.31 to 1.00]; *P* = .049), with a similar trend observed in the other age groups (Data Supplement, Fig S5).

**TABLE 1.** Baseline Demographics and MGUS Characteristics of the Three Study Arms

Characteristic	Arm 1 (n = 1,181)	Arm 2 (n = 1,180)	Arm 3 (n = 1,180)
Age, years, median (range)	69 (42-97)	70 (41-100)	70 (43-100)
Male, No. (%)	644 (55)	638 (54)	643 (55)
Potential follow-up, years^a^	4.8	4.7	4.7
Isotype, No. (%)			
IgA	135 (11.4)	109 (9.2)	140 (11.9)
IgG	599 (50.7)	595 (50.4)	601 (50.9)
IgM	220 (18.6)	233 (19.7)	212 (18.0)
Multiple	85 (7.2)	99 (8.4)	85 (7.2)
Light chain	73 (6.2)	73 (6.2)	73 (6.2)
Light chain—old definition^b^	69 (5.8)	71 (6.0)	69 (5.8)
Abnormal FLC ratio, No. (%)	189 (16.0)	178 (15.1)	180 (15.3)
Kappa	115 (9.7)	98 (8.3)	110 (9.3)
Lambda	74 (6.3)	80 (6.8)	70 (5.9)
M protein, g/dL, median (range)	0.19 (0.01-2.93)	0.19 (0.01-2.60)	0.20 (0.1-2.34)
% with ≥1.5 g/dL	15 (1.3)	15 (1.3)	15 (1.3)
MGUS risk category, No. (%)			
Low risk	432 (36.6)	457 (38.7)	451 (38.2)
Low-intermediate risk	436 (36.9)	417 (35.3)	408 (34.6)
High-intermediate risk	168 (14.2)	158 (13.4)	178 (15.1)
High risk	3 (0.3)	4 (0.3)	1 (0.1)
Light chain	142 (12.0)	144 (12.2)	142 (12.0)

Abbreviations: FLC, free light chain; MGUS, monoclonal gammopathy of undetermined significance.

^a^ As determined by the Kaplan-Meier method with censoring as the event (*P* = .50).

^b^ These participants do not have abnormal FLC testing after applying recently revised normal values for FLC.^16,17^

**FIG 1.**
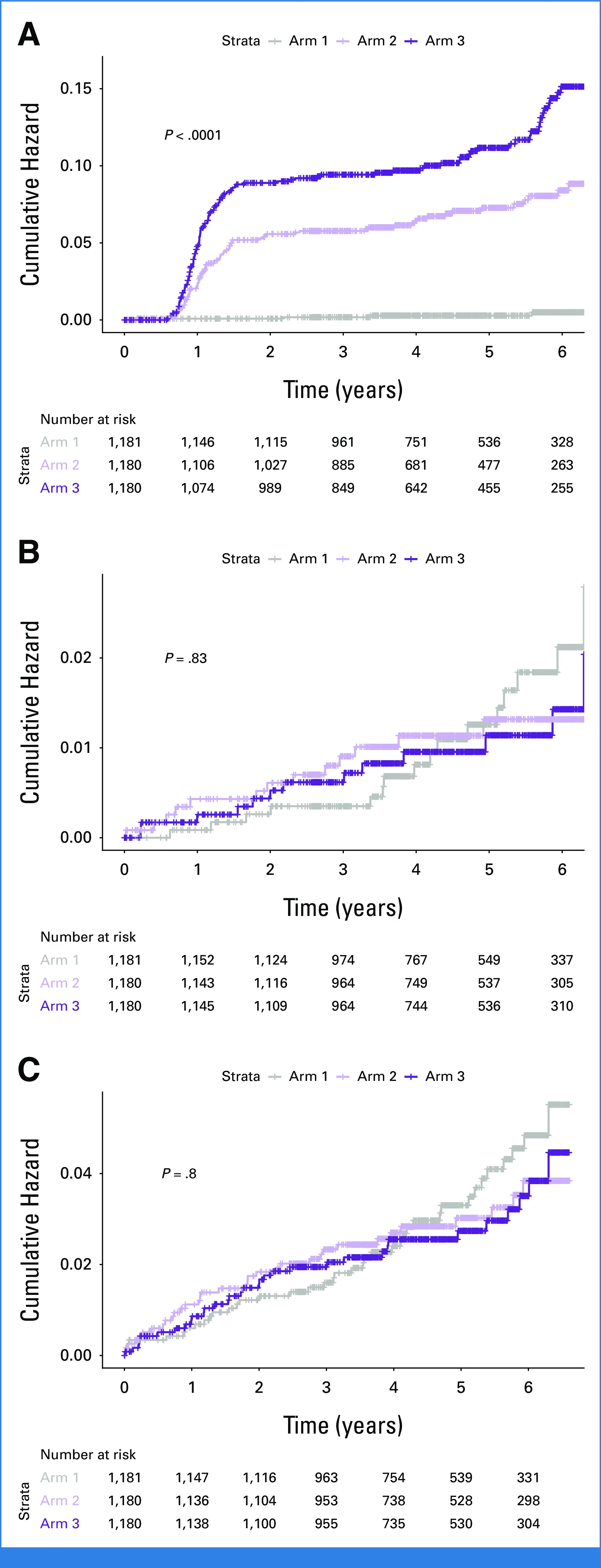
Cumulative hazard plots of progression to (A) smoldering MM, (B) MM, and (C) MM and related clinical disorders in the three arms. The curves were compared using the log-rank test. Note that the scale of the y-axis differs based on the frequency of the end points in question. MM, multiple myeloma.

Treatment for SMM was initiated in 48 participants in the intervention arms and one in the control arm (*P* < .001), of whom 81% 70 years and older. Time from screening to diagnosis was shorter in the intervention arms for both MM (2.4 *v* 3.8 years; *P* = .02) and MM or related malignancies (2.0 *v* 3.0 years; *P* = .02; Data Supplement, Table S3). Symptomatic MM (ie, CRAB-positive) was less frequent in the intervention arms (risk ratio [RR], 0.57 [95% CI, 0.38 to 0.84]; *P* = .03; Fig 2), as were acute presentations of MM requiring hospitalization (RR, 0.54 [95% CI, 0.27 to 1.00]; *P* = .047; Fig 2).

**FIG 2.**
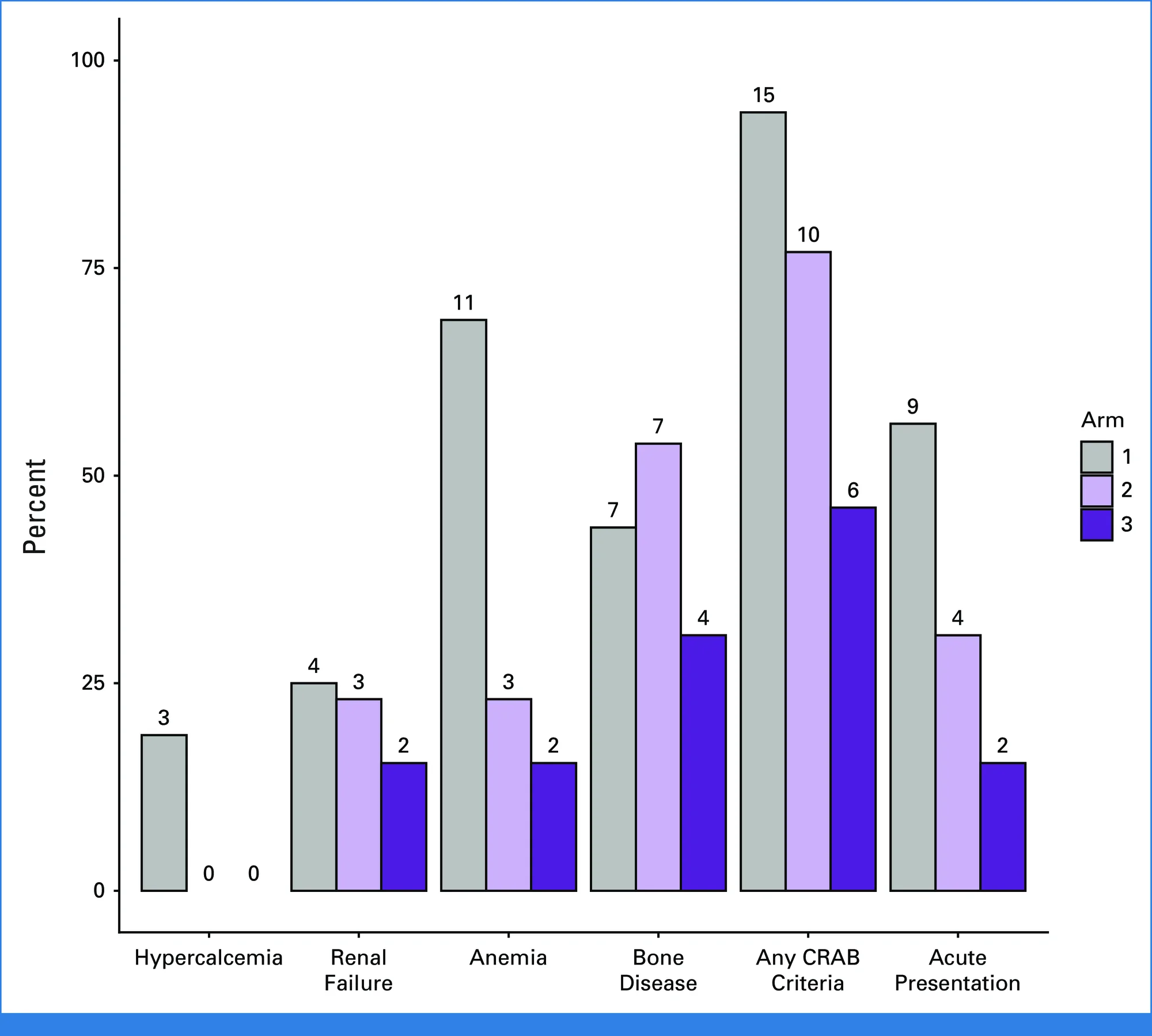
The proportion of active MM patients with different clinical features of MM and acute presentation (defined as requiring hospitalization at diagnosis), by study arm. Numbers above the bars represent the number of cases. MM, multiple myeloma.

Psychological outcomes were assessed using 11,216 questionnaires from 2,593 participants (73%). Throughout follow-up, PHQ-9 (depression) and GAD-7 (anxiety) scores were lower in the intervention arms, with small effect sizes (Cohen's d 0.18-0.23), whereas satisfaction with life scores were similar across groups. Rates of severe depression, anxiety, or life dissatisfaction did not differ before or after 18 months from screening (Data Supplement, Table S6).

## DISCUSSION

In this randomized controlled trial evaluating screening for monoclonal gammopathies including 75,422 participants, population-based screening led to a 27-fold increased detection of SMM and a decreased diagnostic delay for MM and related malignancies by 1 year, before the occurrence of severe end-organ damage. Screening was associated with decreased risk of MM and related malignancies in the age group between 40 and 70 years, which included 81% of those who received early treatment, but not overall. These findings show that screening facilitates earlier detection of MM and may significantly delay or prevent progression. Although a shorter time to diagnosis does not by itself constitute clinical benefit, the findings underscore that screening is a prerequisite for early intervention strategies in high-risk SMM to have a meaningful impact at the population level.

More intensive follow-up led to a 65% increased SMM detection compared with guideline-directed follow-up, probably because of more bone marrow sampling in low-risk MGUS. However, there was no difference in other end points. Although underpowered for this comparison, this study indicates that the intensive approach increases availability of early treatment at the cost of overdiagnosis of SMM. Few participants developed malignancy, and the extensive testing and follow-up in both intervention arms raise questions about the economic viability of screening. Future work should focus on optimizing MGUS care and identifying those who do and do not benefit from follow-up.

Based on more than 11,000 mental health assessments, notification of MGUS was not associated with adverse psychological outcomes. Small differences favoring the intervention arms were clinically insignificant and may reflect bias from clinical contact. This study provides robust evidence that, within a system providing specialized care without individual financial toxicity, MGUS diagnosis through screening does not affect overall mental health.

This study has several strengths, including the randomized design, wide recruitment criteria, high participation rate, and the use of high-quality registry data enabling virtually complete capture of disease progression, including for arm 1. Limitations include relatively short follow-up, reliance on end points that do not directly capture direct benefit and are susceptible to lead-time bias, exclusion of individuals with advanced disease at screening who benefit the most, and a genetically and ethnically homogenous population.

In summary, in this randomized controlled trial evaluating monoclonal gammopathy screening, we found that screening led to a dramatic increase in the detection of SMM and earlier diagnosis of active MM, facilitating timely treatment when end-organ damage has not become clinically apparent. These benefits were not at the cost of psychological distress. Longer follow-up is needed to determine whether early diagnosis translates into improvements in overall and disease-specific survival. Moreover, given the high number of MGUS cases relative to the improvements in early detection, it is unlikely that screening is economically viable in the current paradigm of MGUS care. Nevertheless, our findings highlight how screening, coupled with efficient MGUS care with better risk-adapted approaches, could shift the clinical paradigm from diagnosing MM at advanced, symptomatic stages toward early detection, timely intervention, and potential disease prevention.

## Data Availability

A data sharing statement provided by the authors is available with this article at DOI https://doi.org/10.1200/JCO-25-02771. Current approvals do not allow for sharing of study data. Data may be shared on reasonable request, pending the review of the investigators and the Icelandic National Bioethics Committee.
