# Supplementary material for: Clinical and Psychological Outcomes After Monoclonal Gammopathy Screening: A Population-Based Screening Study and Subsequent Randomized Trial of Follow-Up
Source: J Clin Oncol. 2026 Jul 14;44(29):2781–6. doi: 10.1200/JCO-25-02771 (PMC13668039; doi:10.1200/JCO-25-02771)
Supplement: Supplementary file 1 [file jco-44-2781-s001.pdf]

Clinical and Psychological Outcomes after Monoclonal Gammopathy Screening: A Population-Based Screening Study and Subsequent Randomized Trial of Follow-Up

Supplemental material

1 Supplementary methods 2
1.1 Approvals and ethics 2
1.2 Study participants and screening 2
1.3 Participant follow-up 2
1.4 Measures of psychological outcomes 3
1.5 Statistical analysis 3
2 Supplementary tables and figures 5
Supplementary table 1 5
Supplementary table 2 6
Supplementary table 3 7
Supplementary table 4 10
Supplementary table 5 8
Supplementary table 6 9
Supplementary figure 1 11
Supplementary figure 2 12
Supplementary figure 3 13
Supplementary figure 4 14
Supplementary figure 5 15
3 References 16

1 Supplementary methods

1.1 Approvals and ethics

The study was approved by the Icelandic Bioethics Committee (VSN 16-022, date: 2016-04-26) and the Icelandic Data Protection Agency. The study was preregistered on ClinicalTrials.gov (identifier: NCT03327597). The study protocol has been described in detail elsewhere.[1] All study participants were made aware of the study design with a brief, plain language summary before providing informed consent, and particular care was taken to inform them that they might not be informed of a positive MGUS screening result. All information material given to potential participants was vetted by the Icelandic Bioethics Committee before use. Furthermore, to mitigate any potential inequity created by one treatment arm being blinded to their MGUS status, the study design included biannual interim analyses for differences in overall survival, requiring unblinding if a survival benefit for those in active follow-up would be observed, or after 5-7 years of follow-up when power calculations suggest that a reliable readout for overall survival is possible.

1.2 Study participants and screening

All Icelandic residents born 1975 and earlier who were alive on September 9th, 2016 (n=148,711) were invited to participate. After giving informed consent, participants provided serum samples for the study when they had other blood testing done in the Icelandic Health Service from September 2016 to the end of 2020, and in a targeted blood sample collection drive. Sample accrual was relatively stable over the screening period (Supplemental methods figure 1). Samples were screened by serum protein electrophoresis (SPEP) using capillary zone electrophoresis (CZE; Helena Laboratories, Texas, USA) and FLC assay (Freelite®; The Binding Site Group, Birmingham, UK). For samples with suspected M proteins and/or abnormal FLC results, immunofixation electrophoresis (IFE) was performed. All gels were assessed independently by at least two experienced observers.

1.3 Participant follow-up

Participants with a detectable M protein on IFE or an abnormal FLC assay result (Supplementary methods table 1), were randomized to one of three study arms using block randomization with stratification by M protein concentration ≥1.5g/dL and light-chain MGUS to ensure equal distribution of those rarer presentations of MGUS. Participants with MGUS randomized to Arm 1 were not notified that they had MGUS and continued as if they had not been screened. Those in Arm 2 were followed according to guidelines,[2] which included bone marrow sampling and x-ray imaging at baseline and subsequent follow-up annually with blood tests, including SPEP and FLC assay for non-low risk MGUS and every 2-3 years for low risk MGUS (Supplementary methods table 2). Follow-up for arm 3 was more intensive with baseline bone marrow sampling and whole-body low-dose computed tomography (WBLDCT), and annual follow-up with extended blood tests and urine dipstick for all participants (Supplementary methods table 2). Participants with IgM MGUS in Arms 2 and 3 had bone marrow sampling and a CT scan of the abdomen instead of other imaging. Participants diagnosed with SMM at any time point were managed according to guidelines[2,3] with follow-up every 4-6 months and annual bone marrow sampling and WBLDCT, followed by MRI if WBLDCT was normal. Participants diagnosed with SWM (≥10% bone marrow lymphoplasmacytic cells) had a CT of the neck, thorax, and abdomen performed instead of a WBLDCT. Additional testing was carried out as clinically indicated.

Participants with intermediate and high-risk SMM or active MM were offered participation in an early intervention trial (ClinicalTrials.gov identifier: NCT03815279). Participants in Arm 1 diagnosed with a monoclonal gammopathy outside the study were

invited for follow-up at the study clinic and were eligible for the treatment trial. Participants not eligible for the clinical trial but requiring treatment were referred to the Icelandic health service. Diagnoses of SMM, SWM, active MM, WM, and other lymphoproliferative disorders were acquired from participant follow-up in the study, by review of pathology reports from Landspitali University Hospital, where the diagnoses of these disorders in Iceland are virtually always made, and from the Icelandic Cancer Registry. Reporting of cancer cases in Iceland is mandatory and the registry has been shown to be highly accurate.[4]

1.4 Measures of psychological outcomes

Three validated questionnaires were used to evaluate the psychological effects of screening. The Patient Health Questionnaire 9 (PHQ-9) for symptoms of depression,[5] the General Anxiety Disorder Questionnaire 7 (GAD-7) for symptoms of anxiety,[6] and the Satisfaction with Life Scale (SWLS) to gauge satisfaction with life.[7] All participants were asked to complete these questionnaires at time of consent (before screening) and annually, starting at randomly assigned timepoints, thereafter. Additionally, to capture the immediate psychological impact of receiving an MGUS diagnosis, participants attending the study clinic were asked to complete the questionnaires two weeks after being informed about their MGUS status. This meant that, with the exception of the immediate aftermath of MGUS diagnosis, the questionnaire responses were distributed across the study period, providing broad coverage of timepoints relative to the date of screening.

1.5 Statistical analysis

The primary outcome of this report was a diagnosis of SMM. Secondary outcomes were progression to active MM and active MM and related active malignancies, time to diagnosis of active MM and related disorders, symptom burden at diagnosis, and psychological wellbeing after screening. Participants were followed from the date of screening until progression to: 1) SMM; 2) active MM; and 3) active MM or other related active malignancies with censoring at death. The effect of screening was estimated in an intention-to-treat analysis by comparing Arm 1 (control arm) to Arms 2 and 3 combined (intervention arms). Kaplan-Meier method and a log-rank test were used to compare the rates of progression. Cox regression was then used to quantify any differences between these progression rates and interpreted as average hazard ratios over the study period since non-proportionality was expected. Since younger participants are more likely to be eligible for early treatment and because competing risks of death increase with age, it is probable that the benefits of screening vary by age. Exploratory analyses were therefore performed after stratification into age groups ranging from 40, 45, and 50 years to 70, 75, or 80 years to explore possible effects of the age at screening on outcomes. To evaluate how screening affected diagnostic delay in active MM and related disorders, we compared the time in the control and intervention arms from screening to the diagnosis of active MM and active MM and related disorders in those who received these diagnoses at any time after screening. Symptoms and other clinical features of MM at diagnosis, including requirement for hospital admission at diagnosis, were compared between Arm 1 and Arms 2 and 3 using Fisher exact tests.

Scores on the PHQ-9, GAD-7, and SWLS questionnaires were compared between participants in Arm 1 and those in Arms 2 and 3 using generalized estimating equations with additional covariates of time from screening, age, sex, and interaction between time and arm, as well as a random effect for the participant and time to account for multiple and differing numbers of observations. Using the models, the predicted average score differences between Arms 2 and 3 and Arm 1 were calculated and compared at 6 months, 1 year, and 3 years after MGUS diagnosis. Additionally, the rate of moderate to severe depression and anxiety as well

as rates of life dissatisfaction at any timepoint was compared between Arms 2 and 3 and Arm 1 before and after 18 months post-screening (early vs late effect). Those with a prior diagnosis of MGUS were excluded from all analyses.

Analyses were carried out using R[8] (v. 4.5.1) utilizing the survival[9] and lme4[10] packages.

2 Supplementary tables and figures

Supplementary table 1 Definitions of light-chain monoclonal gammopathy of undetermined significance used for randomization to the iStopMM study, according to level of kidney function. eGFR: Estimated glomerular filtration rate; FLC: Free light chain

	**eGFR > 60**	**eGFR ≤60**
FLC ratio	0.26-1.65	0.37-3.1
	AND	
Raised involved FLC	Kappa >35.85<br>Lambda >38.93	

Supplementary table 2 Clinical assessment, imaging, and laboratory studies included for participants in the different study arms of the iStopMM study. Note that additional testing was ordered if clinical indicated. Recreated from Rögnvaldsson S et al. 2021 Blood Cancer Journal DOI: doi: 10.1038/s41408-021-00480-w.

**Test**	**Arm 2 – Low-risk and LC-MGUS**	**Arm 2 – Non-low-risk**	**Arm 3 – All**	**SMM and SWM**	**MM and WM**
**Physical exam[1]**	First visit	First visit	Each visit	Each visit	At diagnosis
**Blood tests**					
**-SPEP**<br>**-FLC assay**	Each visit	Each visit	Each visit	Each visit	At diagnosis
**-CBC**	First visit	Each visit	Each visit	Each visit	At diagnosis
**-Total calcium**<br>**-Albumin**<br>**-Creatinine**	First visit	First visit	Each visit	Each visit	At diagnosis
**-CRP**<br>**-LDH**<br>**-ß2M**	-	-	Each visit	Each visit	At diagnosis
**-TnT**<br>**-pro-BNP**	-	-	Annually	Annually	At diagnosis
**Bone marrow**					
**-Smear**<br>**-Biopsy**	As clinically indicated	0 months Except if LC	0 and 60 months	Annually	At diagnosis
**Urine**					
**-Protein dipstick**	First visit	First visit	-	-	-
**-UPEP**	If positive dipstick or if previously abnormal	If positive dipstick or if previously abnormal	-	-	-
**-Albumin/creatinine ratio**	-	-	Annually	Annually	At diagnosis
**ECG**	-	-	Annually	Annually	At diagnosis
**Imaging**					
**-WB-LDCT**	-	-	0 and 60 months in LC- and non-IgM	Annually in LC- and non-IgM	At diagnosis of MM
**-Plain X-ray of bones**	As clinically indicated	First visit in LC- and non-IgM	-	-	-
**-CT abdomen**	-	First visit in IgM	0 and 60 months in IgM	Annually in IgM	At diagnosis of WM
**-MRI of bones**	-	-	-	If WB-LDCT is normal	-
**Follow-up**	Every 2-3 years	Annual	Annual	Every 4-6 months	Single visit

SMM: Smoldering Multiple Myeloma. SWM: Smoldering Waldenströms Macroglobulinemia. MM: Multiple myeloma. WM: Waldenströms macroglobulinemia. SPEP: Serum protein electrophoresis. FLC: Free light-chains. CBC: Complete blood count. CRP: C-reactive protein. LDH: Lactate dehydrogenase. ß2M: ß-2-microglobulin. TnT: Troponin T. pro-BPN: pro-brain natriuretic peptide. UPEP: Urine protein electrophoresis. ECG: Electrocardiogram. WB-LDCT: Whole body low-dose computerized tomography. CT: Computerized tomography. MRI: Magnetic resonance imaging. LC: Light chain.

Supplementary table 3 The frequency of progression events by study arm. Mean time to diagnosis was estimated among participants experiencing the event during follow-up. a: Arm 1 vs arms 2 and 3; b: Chronic lymphocytic leukemia and Non-Hodgkin's lymphoma; c: Compared using t-test.

	Arm 1	Arm 2	Arm 3	p[a]
n	1,181	1180	1180	
**Smoldering disorders**				
Smoldering multiple myeloma	4 (0.3%)	78 (6.6%)	125 (10.6%)	
Smoldering Waldenström macroglobulinemia	0	21 (1.8%)	35 (3.0%)	
**Multiple myeloma and related clinical disorders**	40 (3.4%)	35 (2.9%)	35 (2.9%)	
Multiple myeloma	16 (1.4%)	13 (1.1%)	13 (1.1%)	
Waldenström macroglobulinemia	10 (0.8%)	10 (0.8%)	7 (0.6%)	
Other lymphoproliferative disease[b]	14 (1.2%)	12 (1%)	15 (1.2%)	
Amyloidosis	0	1 (0.1%)	1 (0.1%)	
**Mean time to diagnosis (years)**				
Multiple myeloma or related clinical disorder	3.01	2.00	1.93	0.02[c]
Multiple myeloma	3.81	2.02	2.78	0.01[c]

Supplementary table 4 The number of and proportion of progression events by the Mayo MGUS risk score of in each study arm. Note that those with light-chain MGUS are reported separately since they don't conform to the risk score. SMM: Smoldering multiple myeloma; MM: Multiple myeloma

Mayo risk score =	***0***	***1***	***2***	***3***	***Light chain***
**SMM**					
Arm 1	0 (0%)	1 (0.2%)	3 (4.7%)	0 (0%)	0 (0%)
Arm 2	16 (3.0%)	37 (8.5%)	13 (22.4%)	1 (25.0%)	11 (7.6%)
Arm 3	46 (8.6%)	44 (10.0%)	13 (22.8%)	0 (0%)	22 (15.5%)
**MM**					
Arm 1	5 (0.9%)	5 (1.2%)	3 (4.7%)	0 (0%)	3 (2.1%)
Arm 2	3 (0.6%)	3 (0.7%)	4 (6.9%)	0 (0%)	3 (2.1%)
Arm 3	3 (0.6%)	4 (0.9%)	2 (3.5%)	1 (100%)	3 (2.1%)
**MM or related malignancy**					
Arm 1	10 (1.9%)	20 (4.6%)	4 (6.3%)	2 (66.7%)	3 (2.1%)
Arm 2	8 (1.5%)	13 (3.0%)	8 (13.8%)	1 (25.0%)	4 (2.8%)
Arm 3	8 (1.5%)	12 (2.7%)	5 (8.8%)	1 (100%)	7 (4.9%)

Supplementary table 5 Risk stratification of smoldering multiple myeloma (SMM) cases at the time of diagnosis during the study period.

**Risk category**	**n (%)**
Low risk	133 (64%)
Intermediate risk	59 (29%)
High risk	12 (6%)
Missing	3 (1%)

Supplementary table 6 Data on mental wellbeing compared between Arm 1 and Arms 2 and 3. a: Predicted difference based on a general mixed effects linear model. b: Having clinically significant depression, anxiety, or life dissatisfaction at any time during the specified time period. PHQ-9: Patient Health Questionnaire 9. 1: A PHQ-9 score ≥15; 2:

	**Control group (Arm 1)**	**Intervention group (Arms 2 and 3)**	**p**	**Cohens-d**
**n received questionnaire**	951	2114		
n with ≥1 response (%)				
PHQ-9 (Depression)	751 (79.0)	1833 (86.7)	<0.001	
GAD-7 (Anxiety)	749 (78.8)	1832 (86.7)	<0.001	
SWLS (Life satisfaction)	755 (79.4)	1758 (83.2)	0.014	
n responses				
PHQ-9 (Depression)	2586	7967		
GAD-7 (Anxiety)	2591	8053		
SWLS (Life satisfaction)	2649	7439		
**Average differences (95% CI)[a]**				
6 months				
PHQ-9 (Depression)	Ref	-0.7 (-1.1; -0.4)	<0.001	0.18
GAD-7 (Anxiety)	Ref	-0.6 (-0.9; -0.3)	<0.001	0.19
SWLS (Life satisfaction)	Ref	-0.4 (-0.9; 0.2)	0.17	0.06
12 months				
PHQ-9 (Depression)	Ref	-0.7 (-1.1; -0.4)	<0.001	0.18
GAD-7 (Anxiety)	Ref	-0.6 (-0.9; -0.4)	<0.001	0.20
SWLS (Life satisfaction)	Ref	-0.2 (-0.7; 0.3)	0.39	0.04
36 months				
PHQ-9 (Depression)	Ref	-0.7 (-1.1; -0.4)	<0.001	0.18
GAD-7 (Anxiety)	Ref	-0.7 (-1.0; -0.5)	<0.001	0.23
SWLS (Life satisfaction)	Ref	0.4 (-0.04; 0.9)	0.08	0.07
**Clinical outcomes (%)[b]**				
Early (0-18 months)				
Moderate to severe depression[1]	57 (9.6%)	263 (8.3%)	0.34	
Moderate to severe anxiety[2]	26 (4.4%)	106 (3.3%)	0.25	
Life dissatisfaction[3]	27 (4.4%)	138 (5.6%)	0.32	
Late (>18 months)				
Moderate to severe depression[1]	175 (12.4%)	347 (10.4%)	0.05	
Moderate to severe anxiety[2]	60 (4.2%)	130 (3.9%)	0.58	
Life dissatisfaction[3]	84 (5.8%)	178 (5.1%)	0.38	

Supplementary figure 1 The accrual of screening tests in the iStopMM study over the period of screening.

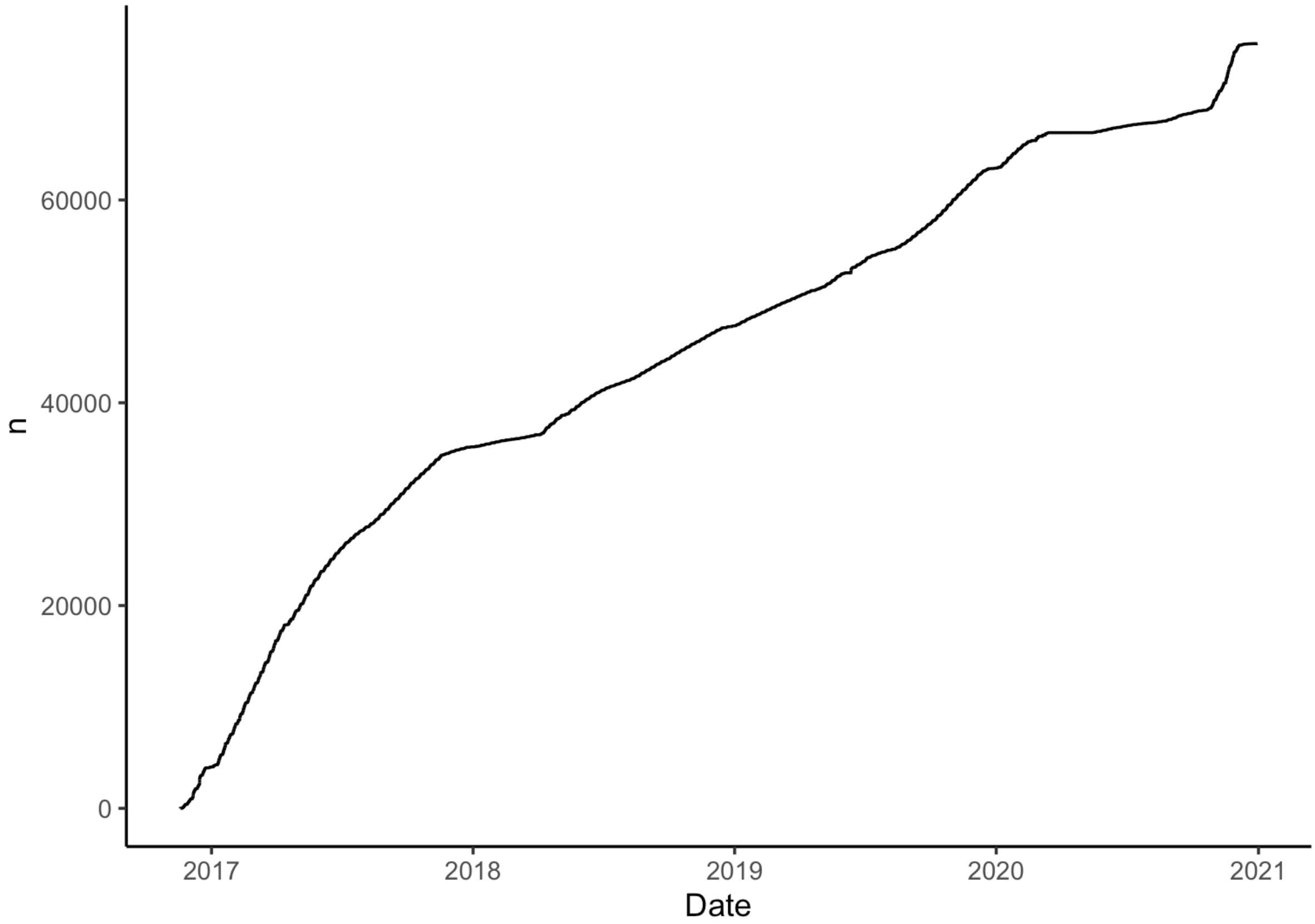

Supplementary figure 2 A CONSORT diagram of the iStopMM study. This includes whether participants visited the study clinic (i.e. received the intervention) at least once. LPD: Lymphoproliferative disorder; MGUS: monoclonal gammopathy of undetermined significance

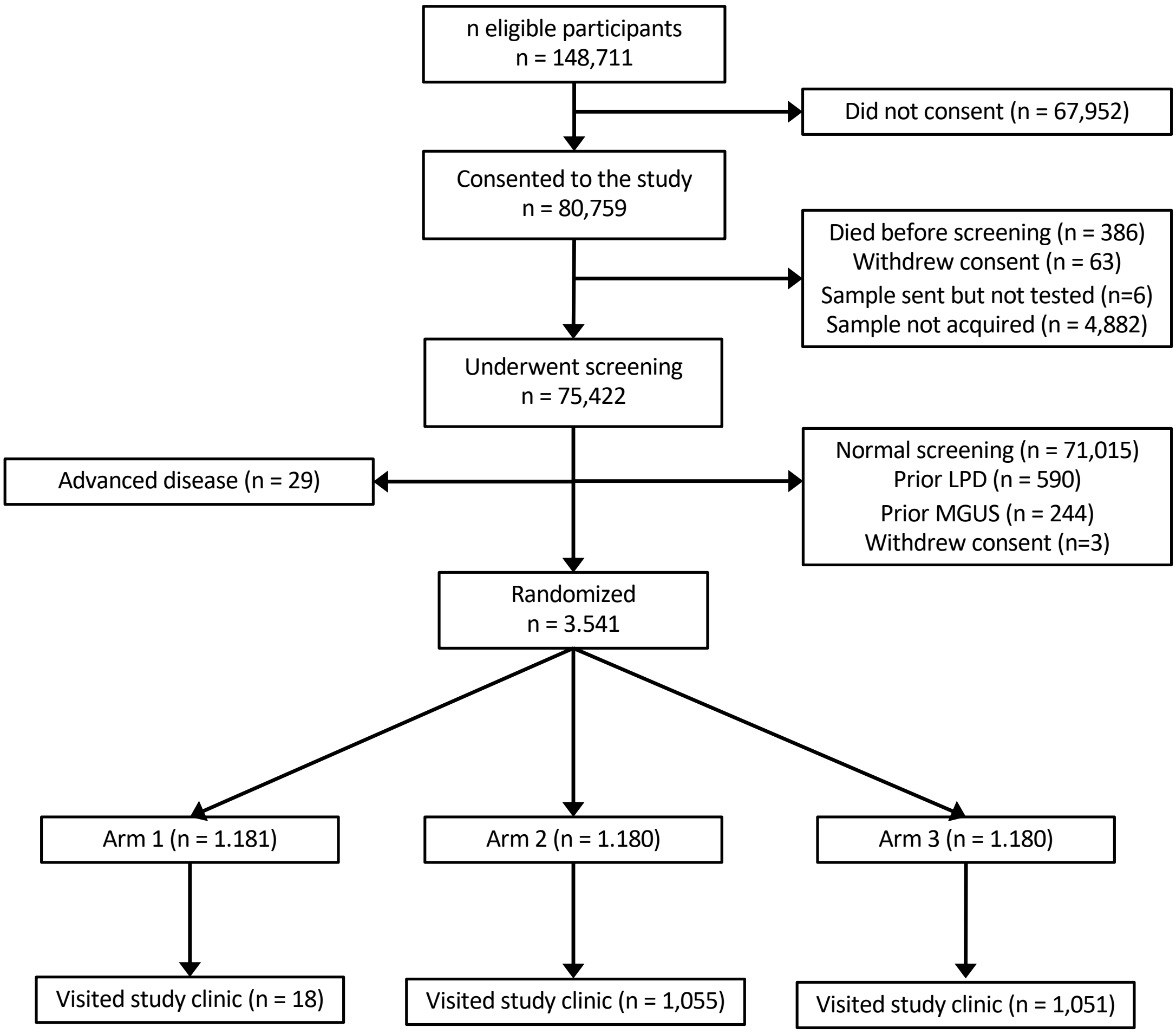

Supplementary figure 3 Cumulative hazard plots for A: Smoldering multiple myeloma; B: Multiple myeloma, and C: Multiple myeloma or related clinical disorders. Comparisons between for the combined Arms 2 and 3 (purple) and Arm 1 (grey). The curves were compared using log-rank tests. Note that scales of the y-axes differs by outcome due to the different incidence of the outcomes.

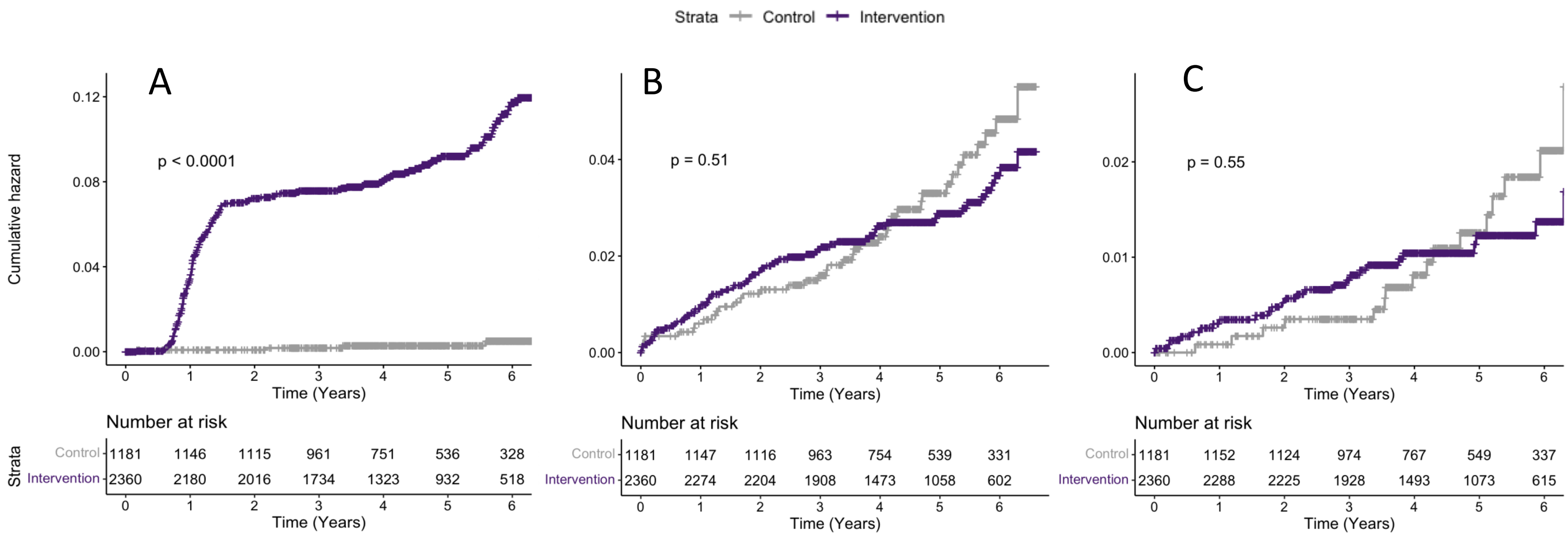

Supplementary figure 4 Cumulative hazard plots CRAB positive MM stratified by Arm and by control/intervention arms.

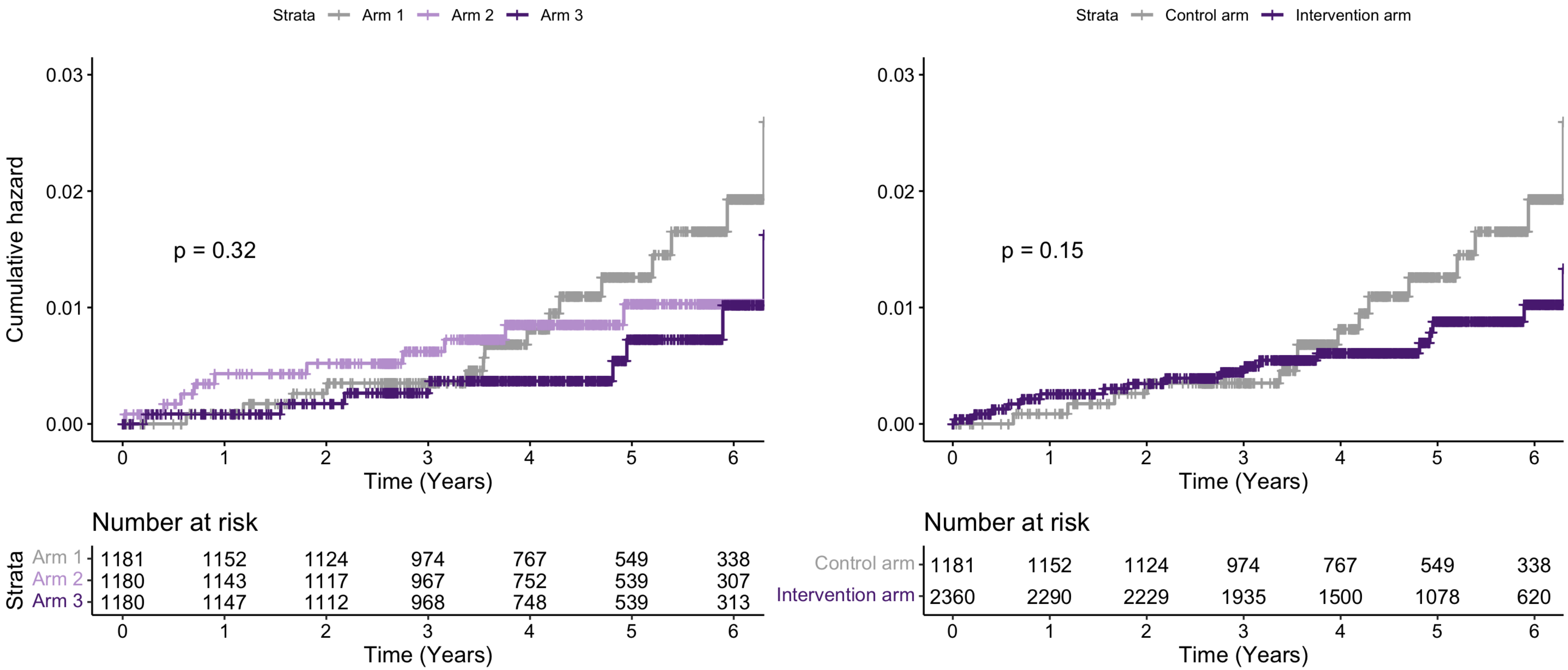

Supplementary figure 5 Cumulative hazard plots for progression to multiple myeloma or related malignancies according to whether participants were randomized to the intervention arms (Arms 2 and 3) or the control arm (Arm 1), when limiting the cohort to different reasonable target populations. The columns represent different lower ends and the rows different upper ends of the age group.

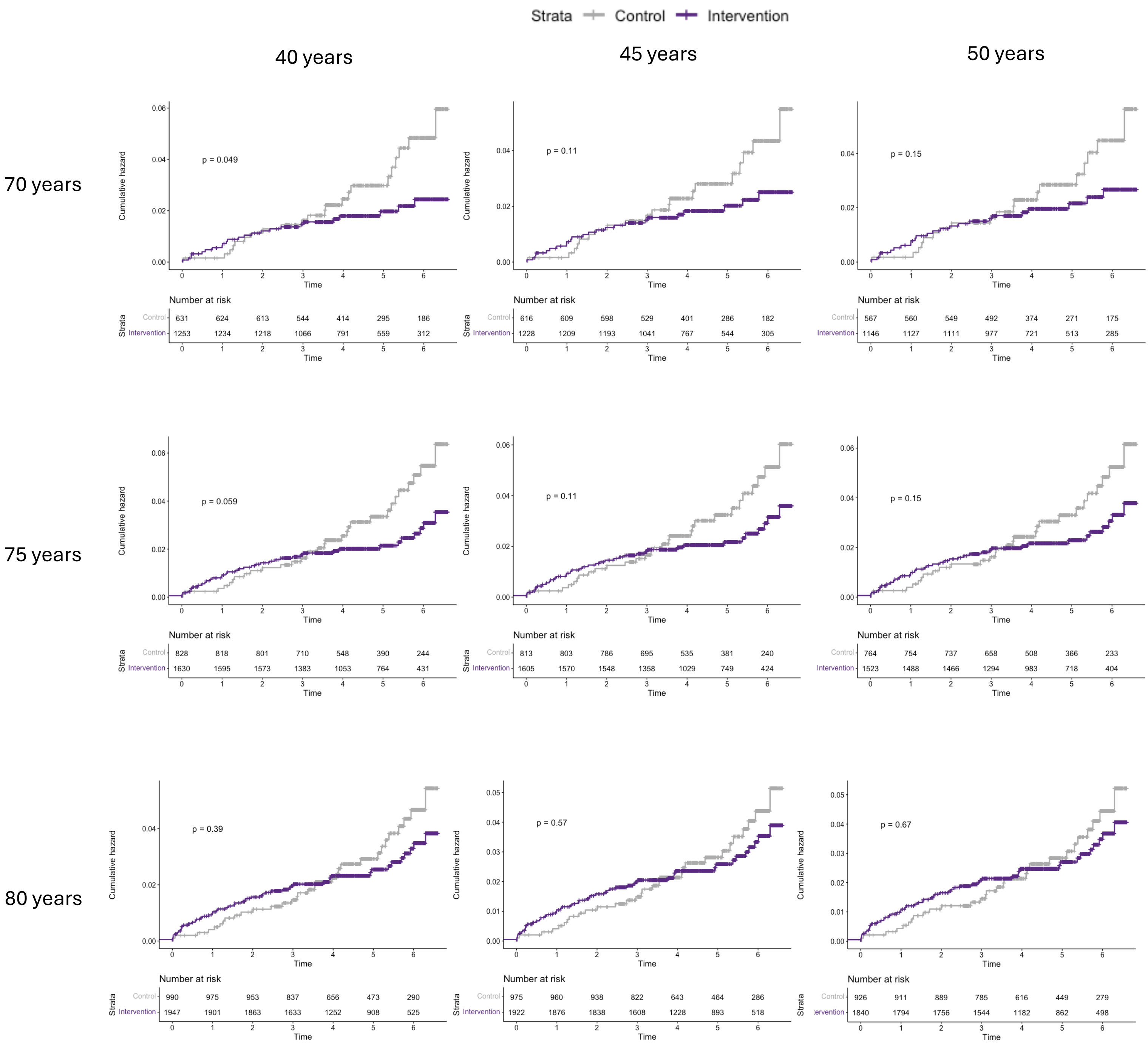

3 References

1. Rögnvaldsson S, Love TJ, Thorsteinsdottir S, et al: Iceland screens, treats, or prevents multiple myeloma (iStopMM): a population-based screening study for monoclonal gammopathy of undetermined significance and randomized controlled trial of follow-up strategies. Blood Cancer Journal 11:94, 2021
2. Kyle RA, Durie BGM, Rajkumar SV, et al: Monoclonal gammopathy of undetermined significance (MGUS) and smoldering (asymptomatic) multiple myeloma: IMWG consensus perspectives risk factors for progression and guidelines for monitoring and management. Leukemia 24:1121-1127, 2010
3. Rajkumar SV, Dimopoulos MA, Palumbo A, et al: International Myeloma Working Group updated criteria for the diagnosis of multiple myeloma. The Lancet Oncology 15:e538-e548, 2014
4. Sigurdardottir LG, Jonasson JG, Stefansdottir S, et al: Data quality at the Icelandic Cancer Registry: Comparability, validity, timeliness and completeness. Acta Oncologica 51:880-889, 2012
5. Martin A, Rief W, Klaiberg A, et al: Validity of the brief patient health questionnaire mood scale (PHQ-9) in the general population. General hospital psychiatry 28:71-77, 2006
6. Spitzer RL, Kroenke K, Williams JB, et al: A brief measure for assessing generalized anxiety disorder: the GAD-7. Arch Intern Med 166:1092-7, 2006
7. Diener E, Emmons RA, Larsen RJ, et al: The Satisfaction With Life Scale. J Pers Assess 49:71-5, 1985
8. R Core Team: R: A language and environment for statistical computing. 2013
9. Therneau TM: A Package for Survival Analysis in R, 2023
10. Bates D, Mächler M, Bolker B, et al: Fitting Linear Mixed-Effects Models Using lme4. Journal of Statistical Software 67:1 - 48, 2015
