# Supplementary material for: Clinical and Psychological Outcomes After Monoclonal Gammopathy Screening: A Population-Based Screening Study and Subsequent Randomized Trial of Follow-Up
Source: J Clin Oncol. 2026 Jul 14;44(29):2781–6. doi: 10.1200/JCO-25-02771 (PMC13668039; doi:10.1200/JCO-25-02771)
Supplement: Supplementary file 2 [file jco-44-2781-s002.pdf]

ASCO Journals Data Sharing Statement Questionnaire

**1. Manuscript Title**	Clinical and Psychological Outcomes after Monoclonal Gammopathy Screening: A F
**2. First Author Last Name**	Rögnvaldsson
**3. Does your manuscript use ONLY data from a publicly available database (eg, SEER, Medicare)?** If "Yes," please specify the database; no additional information is needed. If "No," please complete questions 4-17.	No
**4. Will the data collected for your study be made available to others?** (If "No," enter context for your decision)	Partially. Individual-level participant data cannot be shared under current ethical approvals but may be shared pending investigator and ethical approval. Aggregated data my be made available on reasonable request.
**5. List which data are available**	Aggregated summary data suitable for meta-analysis or similar secondary research
**6. Additional information about the data** (Enter "None," if applicable)	None
**7. List how or where the data can be obtained** (e.g., e-mail address, URL, or other repository)	Requests should be directed to the corresponding author
**8. List beginning and end dates that data will be available** (e.g., MM/DD/YYYY to MM/DD/YYYY)	Upon publication, indefinately. (subject to ethical approcal and study team capacity)
**9. List any supporting documents** (Enter "None," if applicable)	None
**10. Enter additional information about supporting documents** (Enter "None," if applicable)	None

**11. How or where can supporting documents be obtained?** (e.g., e-mail address, URL, or other repository)	N/A
**12. List beginning and end dates that supporting documents will be available** (e.g., MM/DD/YYYY to MM/DD/YYYY)	N/A
**13. Indicate to whom data will be available** (Enter “U/K” for unknown; “N/A” for not applicable)	Qualified researchers with a scientifically valid request and appropriate institutional affiliation.
**14. Indicate for what type of analysis or purpose** (Enter “U/K” for unknown)	Meta-analysis or similar secondary research purposes. Individual-level data requests will be considered on a case-by-case basis pending ethical approval.
**15. Indicate by what mechanism** (Enter “U/K” for unknown)	Direct request to the corresponding author pending revew of the iStopMM study investigators and the Icelandic national bioethics comittee
**16. Enter any other restrictions** (Enter “None,” if applicable)	As described above
**17. Enter any additional information** (Enter “None,” if applicable)	None

(Optional) If you have included or would like to include a Data Sharing Statement in addition to the above, please provide on the following page.

Data Sharing Statement
