# Supplementary material for: Clinical and Psychological Outcomes After Monoclonal Gammopathy Screening: A Population-Based Screening Study and Subsequent Randomized Trial of Follow-Up
Source: J Clin Oncol. 2026 Jul 14;44(29):2781–6. doi: 10.1200/JCO-25-02771 (PMC13668039; doi:10.1200/JCO-25-02771)
Supplement: Supplementary file 3 [file jco-44-2781-s003.pdf]

HÁSKÓLI ÍSLANDS

Study Protocol

ICELAND SCREENS, TREATS, OR PREVENTS MULTIPLE MYELOMA (iStopMM)

A population based screening study of monoclonal gammopathy of undetermined significance

Principal Investigator	Sigurður Yngvi Kristinsson, MD, PhD, Professor<br>University of Iceland, Medical Faculty, Vatnsmyrarvegur 16, 101 Reykjavik
VSN registration number	16-022
Funding	Black Swan´s Research Initiative<br>European Research Council<br>Rannis
Study Center	iStopMM Clinic, Skogarhlid 8, 105 Reykjavik
Version number	2.0
Release date	2017-04-12
Independent Data Monitoring Committee	Signý Vala Sveinsdóttir<br>Ubaldo Benitez Hernandez<br>Inga Sigurrós Þráinsdóttir
Collaborators	Þorvarður Jón Löve, MD, PhD, Associate Professor Rheumatologist<br>Ola Landgren, MD, PhD, Professor at Memorial Sloan Kettering Cancer Center<br>Stephen Harding, PhD, Biochemistry – The Binding Site<br>Ísleifur Ólafsson, MD, PhD, Head of Clinical Biochemistry and Laboratory at Landspitali University Hospital<br>Hlíf Steingrímsdóttir, MD, Director of Internal Medicine at Landspitali University Hospital<br>Kristján Oddsson, MD PhD, CEO of the Icelandic Cancer Society<br>Andri Steinthor Bjornsson, PhD, Professor of Clinical Psychology at the University of Iceland<br>Tinna Laufey Ásgeirsdóttir, PhD in Health Economics and Professor at the University of Iceland<br>Ásbjörn Jónsson, Head Department of Radiology at UI Hospital<br>Brynjar Viðarsson, Haematologist at UI Hospital<br>Páll Torfi Önundarson, Professor and Head of Laboratory Haematology at UI Hospital<br>Bjarni Agnarsson, Professor and Pathologist at UI Hospital<br>Margrét Sigurðardóttir, Pathologist at UI Hospital<br>Ragnar Danielsen, Cardiologist

	Runólfur Pálsson, Head of Nephrology Department at UI Hospital<br>Ólafur Skúli Indriðason, Nephrologist at UI Hospital<br>Einar Stefán Björnsson, Professor and Head of Internal Medicine at UI Hospital<br>Elías Ólafsson, MD, Professor, Head of Neurology at UI Hospital<br>Ingunn Þorsteinsdóttir, MD PhD, specializes in clinical biochemistry, UI Hospital<br>Sveinn Guðmundsson, MD PhD, Director of Blood Bank, UI Hospital<br>Anna Margrét Halldórsdóttir, MD, PhD, Consulting Physician, Department of Transfusion Medicine<br>Elías Eyþórsson, MD and PhD student<br>Sæmundur Rögnvaldsson, PhD Student
Collaboratoring institutions	University of Iceland<br>Landspitali, University Hospital<br>Icelandic Cancer Society<br>The Icelandic Heart Association<br>deCode Genetics<br>International Myeloma Foundation/Black Swan Research Initiative<br>The Binding Site<br>Memorial Sloan Kettering Cancer Center<br>The Icelandic Blood Bank

Version	Date	Summary of Changes
1.0	2017-04-12	Initial
2.0	2017-12-22	Minor amendments made to improve coherence of protocol, schedules of procedures and flow charts. Timing of procedures is more detailed. Tracks are more defined and differentiated and therefore the number of tracks has grown. Collaborators and CMD members added.

TABLE OF CONTENT

1.Background **5**
1.1. State of the art **5**
1.2. Etiology **6**
1.3.“Undetermined” significance? **7**
1.4. Progression of precursor states **7**
1.5. Treatment of multiple myeloma precursor states **10**
1.6. Screening **10**

2. Objectives **12**
2.1. Specific aims **12**

3. Methodology **13**
3.1. Study design **13**
3.2. Study population **13**
3.2.1. Computerized database 13
3.2.2. Pilot Project 14
3.2.3. Registries 14
3.2.4. Specific risk groups 14
3.3. Protocol design **14**
3.3.1. Clinical team meeting 15
3.3.2. Clinical study center 15
3.3.3. Groups 15
3.3.4. Tracks 15
3.4. Screening **16**
3.4.1. Blood sampling 16
3.4.2. Electrophoresis and free light chain analyses 17
3.5. Abnormal non-MGUS group **17**
3.6. MGUS group **17**
3.6.1. Inclusion criteria: 18
3.6.2. Exclusion criteria: 18
3.6.3. Arm 1 18
3.6.4. Arm 2 18
3.6.5. Arm 3 18
3.6.6. Randomization and blinding 19
3.7. Normal group **19**
3.7.1. Controls 19
3.7.2. Blinding 19
3.8. Onset of new symptoms **19**
3.9. Outcomes measures in RCT **20**
3.10. Medical history and physical examination **20**
3.11. Assessments of quality of life, anxiety and depression **20**
3.12. Assessment of cost-effectiveness **21**
3.13. Assessment of cardiac function **21**
3.14. Assessment of peripheral neuropathy **21**
3.15. Assessment for hepatomegaly **21**
3.16. Germline genetics **21**
3.17. Flow cytometry **21**
3.17.1 Study population 21
3.17.2. Samples studied by flow cytometry 22
3.17.3. Protocols and panels 22
3.18. Biobanking **22**
3.18.1 Bone marrow 22

3.18.2. Peripheral blood 22
3.18.3. Urine 22

4. Monitoring 23
4.1. Interim analyses 23
4.2. Data monitoring committee 23
4.3.Clinical monitoring 23

5. Data management 23
5.1. Data management system 23
5.2. Data storage 24
5.3. Identifier encryption 24

6. Statistical analysis 24

7. Ethics 24

8. References 25

1.BACKGROUND

1.1. State of the art

Four percent of individuals over the age of 50 years have a monoclonal gammopathy, which is associated with a 1-2% annual risk of malignant transformation and reduced overall survival. Currently we are unaware of the underlying causes of monoclonal gammopathies, we cannot predict which individuals with precursor states will progress to full-blown malignancy, there is no evidence from prospective studies to guide clinicians and patients for the optimal follow-up, the impact of hereditary factors, genetics, imaging, and other molecular markers on transformation is unknown, nor do we have scientific evidence for screening. Thus all individuals with precursor states are identified out of pure coincidence because of another unrelated sickness. Our proposed study will be the first screening study on monoclonal gammopathy of undetermined significance (MGUS) in a whole nation and the first prospective randomized clinical trial with the aim of evaluating the impact of screening and the best follow-up strategy for MGUS.

Monoclonal gammopathies are disorders characterized by a spike in a homogeneous immunoglobulin (the M-protein) in serum (SPEP) or urine (UPEP) protein electrophoresis, or an abnormal kappa to lambda ratio on free light chain (FLC) analyses, and can be detected by a simple test, and arise from the proliferation of an abnormal clone of a single plasma cell precursor.[1] Multiple myeloma (MM) is the archetype of a malignant monoclonal plasma cell disorder. Once it was observed that a substantial proportion of benign gammopathies develop into MM, the now universally accepted term *monoclonal gammopathy of undetermined significance (MGUS)* was introduced to describe the disorder.[2]

MM is a neoplasm characterized by the proliferation and accumulation of malignant plasma cells in the bone marrow that lead to the overproduction of M-protein in the serum or urine. In the Western world, the annual age-adjusted incidence for MM is 4.8 to 8 per 100,000.[3] The median age at diagnosis is approximately 70 years.[4] A total of 40 individuals are diagnosed with MM or related lymphoproliferative disorder every year, thus after 5 years of follow-up in our study approximately 200 participants will have progressed.

Myeloma related end-organ or tissue impairment resulting from this disorder include the classic "CRAB" symptoms of hypercalcemia, renal insufficiency, anemia, and lytic bone lesions and the less classical symptoms of hyperviscosity, amyloidosis, and recurrent bacterial infections.[5] In addition, in the recently updated diagnostic criteria, MM now includes biomarkers and imaging in the diagnostic criteria.[1] MM remains incurable, however, in recent years, several new therapeutic agents with novel mechanisms of action have been approved for MM such as immunomodulatory agents and proteasome inhibitors, and few cancers have experienced more rapid treatment development in recent years.[6] This has led to a significant improvement in survival of MM in the general population,[4, 7-10] with the current median overall survival of more than 6 years (compared to 2-3 years in the 1990s).[11, 12] **Now, the evidence is emerging to consider population screening to identify subjects at risk for**

MM and follow-up studies to start treatment in an earlier phase of the disease.

MGUS denotes the presence of an M-protein on electrophoresis in individuals without evidence of MM, Waldenström macroglobulinemia, amyloidosis or other lymphoproliferative diseases. MGUS is characterized by the following: a serum M-protein concentration less than 30 g/L; less than 10% clonal plasma cells in the bone marrow; little or no M-protein in the urine; absence of the CRAB criteria.[1] Light-chain MGUS (LC-MGUS) is recently described and is based on FLC analysis, and is defined as a pathological FLC ratio (outside reference range 0.26-1.65) in combination with an increased concentration of the light-chain concerned (f-kappa>19.4 or f-lambda >26.3), without evidence of MM, lymphoproliferative diseases, amyloidosis or classical MGUS.[13]

There are 3 types of MGUS with distinct natural histories: non-IgM-(IgG or IgA)-MGUS, IgM-MGUS and LC-MGUS.[13, 14] While IgG and IgA- and LC-MGUS typically progress to MM or develop light-chain-amyloidosis, IgM-MGUS cases more often progress to lymphomas.

The prevalence of MGUS is highly dependent on age and is reported to be found in approximately 5-10% of those older than 70 years which makes it a very attractive condition to study in order to understand plasma cell diseases.[15, 16] Data from the Mayo Clinic suggests that MGUS is present in about 4.2% of the general Caucasian population aged 50 years and older and is predominantly diagnosed incidentally.[13, 15] We recently performed a large, population-based screening cohort study,[17] and included 5,764 Icelanders born 1907-1934 in Iceland. We found the prevalence of MGUS to be 5.3% and LC-MGUS was 1%, thus we confirm that Iceland has a similar prevalence as other western countries, making it suitable for nationwide studies, especially since its population is approximately 340,000 with few people emigrating and a government-maintained health care regardless of socioeconomic status. **The results can be used to assess whether population wide screening is beneficial on a European level but also to determine the disease mechanisms.**

Similar to MGUS, smoldering MM is a precursor condition to MM and is defined by the clinical parameters of M-protein ≥ 30 g/L or bone marrow plasma cells ≥ 10%.[1] We recently found, based on all smoldering MM patients diagnosed in Sweden 2008-2011, that smoldering MM accounts for 14.4% of all MMs the age-standardized incidence of smoldering MM was 0.44 cases per 100,000 persons.[18] Smoldering MM is therefore included in our studies.

1.2. Etiology

The etiology of MGUS is unknown. We have shown that there is a familial aggregation in MGUS and related diseases.[19-24] Additionally, differences in racial disparity patterns have been observed in MGUS (with the risk of Africans and African-Americans being approximately twice as that if whites), suggesting genetic or environmental causes.[25, 26] The genetic basis of inherited MM and MGUS susceptibility is incompletely understood. Recent genome-wide association studies have identified eight common sequence variants that associate with MM, and account for an estimated 13% of the familial risk.[27-29] We

recently performed a study and identified a new MM risk locus at the ELL2 gene, which encodes a stoichiometrically limiting component of the super-elongation complex that drives secretory-specific immunoglobulin mRNA production and transcriptional regulation in plasma cells.[30] However, these known variants account for a minority of MM and MGUS cases and further work is needed. In addition, the clinical impact of these variants on progression from MGUS to MM is completely unknown. Interestingly, we found that the MM risk allele at ELL2 also associated with reduced levels of immunoglobulin A and G in healthy subjects and, with an increased risk of bacterial meningitis.[30] There is further epidemiological evidence for an association between chronic antigen stimulation and risk of MM, MGUS and lymphoproliferative diseases, and individuals with prior history of infections and autoimmune diseases have been shown to be at increased risk of these disorders.[31-35] How prior history of infections and autoimmune disease affects progression to MM is not known and needs to be studied. Additionally, the interaction between environmental and genetic factors is not known and could be of relevance. One of the challenges in identifying risk factors has been limited number of MGUS individuals, lack of information on genetic factors, and the relationship and possible synergism between hereditary and environmental factors. **Our plan is to evaluate the impact of germline genetic factors in this disease, which has never been done MM or related diseases.**

1.3.“Undetermined” significance?

Since the term “undetermined significance” was introduced,[2] we and others have shown, in a number of clinical and screening studies that individuals with MGUS have not only an increased risk of progression to MM, lymphoproliferative diseases, and amyloidosis, but also other diseases, such as fractures[36-41], infections[42, 43], thrombosis[44, 45], non-lymphoproliferative malignancies[46] and inferior survival compared to non-MGUS individuals.[47-51] Additionally, we found increased risk of death due to malignant and non-malignant disorders.[52] Therefore, we should probably rethink the term “undetermined significance”, as a lot of data have emerged since it was introduced almost 40 years ago, indicating the clinical significance of MGUS. **It has become clear in recent years that MGUS is of major significance and its impact on a national level needs to be established in a population-based prospective study. Our plan is to provide the evidence for the significance of MGUS on a population-based level that will have important consequences in other counties as well.**

1.4. Progression of precursor states

The big research question in precursor states is to identify those that progress and might benefit from intervention. Current risk models for progression include different markers, and are thus not integrated with one another, have limited direct correlation to biology, and cannot reliably predict progression in individuals with MGUS.

The risk of progression in smoldering MM is estimated to be an average of 10% annually for the first 5 years following diagnosis (51% risk of progression at 5 years), then decreasing to 3% annually for the next 5 years and 1% annually for the following 10 years.[53]

Two large independent studies indicate that MGUS always precedes MM.[54, 55] The average risk of progression is estimated to be 1.5% per year.[47, 56, 57] However, there is considerable variation in the risk of progression, and differentiating low-risk individuals, who may not need further follow-up, from high-risk individuals, who may warrant close monitoring or enrolment in early intervention studies, is a challenge. Models have been published for risk stratification of MGUS individuals.[56, 58] Risk factors included in a Mayo Clinic model are non-IgG isotype, M-protein concentration ≥15 g/L, and an abnormal serum FLC ratio.[56] Risk factors in the Spanish study group (PETHEMA) model are based on multiparametric flow cytometry and include the presence of ≥95% abnormal plasma cells and aneuploidy.[58] **Similarly, two prognostic scores exist for smoldering MM, however a recent study found almost 30% discordance between the models, emphasising the need for prospectively validated models in MGUS or smoldering MM.[59]**

Importantly, the optimal imaging technique is not established and modern radiological examinations (such as CT) have not been evaluated in large prospective studies. The current standard for skeletal imaging in individuals with monoclonal gammopathies is the conventional skeletal X-ray. It has some major limitations, including limited sensitivity and specificity, and it is time consuming for patients and observers.[60] CT scans have been shown to be superior however there are some concerns regarding radiation exposure, particularly in individuals with MGUS. Recently, whole body low-dose multidetector row-CT have been studied and have been shown to detect small lesions, are much faster than standard radiology, can show soft tissue disease and has higher sensitivity and specificity than conventional X-ray.[60-62] It has been shown to be more sensitive than MRI to detect small lesions.[63] MRI has some benefits, for example to no radiation and is able detect marrow infiltration, however this is not of major importance as bone marrow biopsies are taken and in addition it is more time consuming. The radiation exposure using low-dose CT is only 1.7 times more than standard X-ray, whereas conventional whole body CT is 10.6 times higher radiation, with very good quality.[64] **Therefore, low-dose CT could be the optimal radiological examination for MGUS individuals, and this needs to be addressed in a prospective study.**

At this time there is no way to prospectively determine which individuals will follow which course at the time of initial diagnosis. There are no prospective studies to guide management of individuals with MGUS, however expert opinions suggest, depending on the individual patient's clinical risk score, life-long monitoring of MGUS individuals to detect progression to MM or related disorders.[65, 66] The impact of annual monitoring on the outcome of individuals who eventually develop MM or related diseases is unclear. **There is a great unmet need for a prospective trial to evaluate the most optimal follow-up strategy that is effective, and minimizes the need for unnecessary work-up. Our plan is to perform a nationwide screening study, and consequently a randomized clinical trial (RCT) to evaluate the optimal follow-up strategy.**

To evaluate the impact of MGUS follow-up, we recently performed a large population-based study on more than 14,000 MM patients diagnosed in Sweden 1976-2005, with follow-up through 2007.[67] We compared survival in those with a known prior MGUS compared to those without prior knowledge. We found a

total of 394 MM patients had previously been diagnosed with MGUS and MM patients with prior knowledge of MGUS had significantly 14% decreased risk of death (HR=0.86; 95% CI 0.77- 0.96) than MM patients without prior knowledge of MGUS (Figure). Results were similar when restricted to patients diagnosed with MM after 1996, with MM patients with prior knowledge of MGUS having significantly (HR=0.81; 95% CI 0.70-0.93) better survival than MM patients without prior knowledge of MGUS. Our findings are consistent with a prior smaller study including 116 MM patients seen at the Mayo Clinic between 1973 and 2004 showing that low-risk MGUS individuals are more often diagnosed with serious complications compared to high-risk MGUS individuals.[68] Also a similar finding was observed in another large cohort study from USA.[69] Interestingly, in our study, we found that among MM patients with prior knowledge of MGUS, low M-protein concentration (<5 g/L) at MGUS diagnosis (low-risk) was associated with poorer MM survival (HR, 1.86; 95% CI, 1.13-3.04). This may be reflective of current expert opinion suggesting less frequent monitoring of MGUS individuals with lower M-protein concentrations (low-risk individuals). We speculate that the reason for the prolonged survival observed in our study, most likely reflects the fact that MGUS individuals are evaluated more often for signs of MM progression and may be diagnosed and started on anti-myeloma therapy at an earlier stage. **This argues for early treatment approaches in MM,[70] and raises the question whether systematic screening for MGUS should be initiated.** Alternatively, those with more aggressive disease may have a shorter duration of MGUS and are less likely to be diagnosed and captured during the MGUS state. This is a very important issue, especially in the current times when there is a paradigm change in the way we think of these disease and its management, with novel drugs proving to benefit even the asymptomatic patients (see below). This can only be properly analyzed in a prospective study like our proposed study.[71]

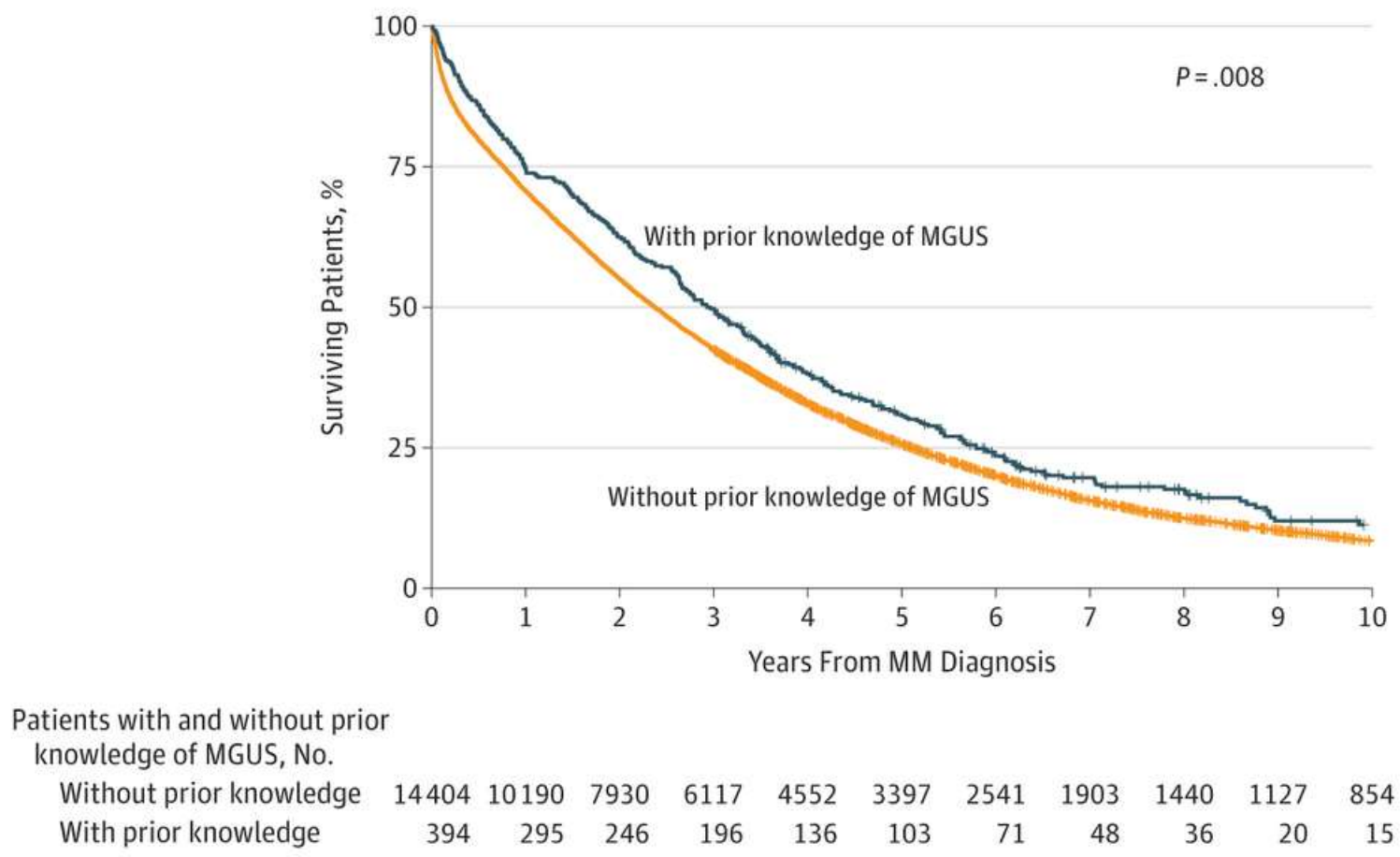

Figure. Survival among MM patients with and without prior knowledge of MGUS

1.5. Treatment of multiple myeloma precursor states

As discussed above, it has become relevant to consider preventive management of patients with smoldering MM and possibly high-risk MGUS.[72] While earlier studies using older and less effective treatment options did not demonstrate a survival benefit from treating smoldering MM,[73-75] recent prospective RCT using lenalidomide and dexamethasone versus no treatment in high-risk smoldering MM showed that the risk of progression to MM was significantly lower and importantly, overall survival was significantly longer in lenalidomide-dexamethasone treated patients.[70] Several RCT are ongoing validating this important finding that suggests a paradigm change in the management of smoldering MM. Very recently, the NCI group published a study on 12 smoldering MM patients with carfilzomib, lenalidomide and dexamethasone.[76] This trial showed that all patients reached minimal residual disease negativity, suggesting that this might be the future reference for standard of care. Very few studies have been performed to treat individuals with MGUS, which is logical as most MGUS individuals will not progress. One study group, however, treated 26 MGUS individuals with oral curcumin, the most active component of the commonly used Indian spice turmeric, and observed a decrease in M-protein.[77] Interestingly, a recent cohort study, including 2,003 MGUS individuals with diabetes mellitus, found that those that were on metformin (for diabetes mellitus) had a significantly lower risk of MM transformation than non-metformin users.[78]

It is very likely that in the coming years, with more potent therapies entering the therapeutic arsenal with fewer side effects, treatment of precursor states will be shown to improve survival and QoL in individuals with smoldering MM and MGUS. These individuals are, by definition, asymptomatic and therefore they will be diagnosed either as a coincidence due to work-up for an unrelated medical condition or as a part of a screening program. **Given that the literature is growing and favouring treatment of high-risk asymptomatic patients, one major challenge is the fact that most of these individuals will remain undiagnosed.** Currently most expert guidelines do not recommend treating high risk smoldering MM, however we envision that in the near future, there will be a paradigm change with regards to management of these patients. As soon as another RCT shows difference in overall survival favouring active treatment against therapeutic abstinence, there will be a major shift in treatment strategies throughout the world. Many authorities consider that early treatment is the way to go if we are aiming to cure MM.[79] **With that in mind, our ambitious program will identify all these individuals in the Icelandic population, and can therefore capture a malignant transformation earlier and thus being able to offer treatment and hopefully cure for these patients as the evidence emerges.** We predict, based on ongoing studies and recent results,[70, 76] that these advances will probably emerge within the next few years.

1.6. Screening

Screening for a disease is not uncontroversial.[80-82] Several aspects need to be taken into account, such as clinical benefit of early detection, potential harm, and cost. These special circumstances to invite individuals without knowledge of a disease in question for screening can have desirable and undesirable

consequences. One important topic is the anxiety and distress associated with the knowledge of a precursor disease. This has not been studied at all in MGUS and the literature on this issue is very limited in screening for cancer in general, however there are studies on colorectal, prostate, ovarian, and breast cancer that suggest that the burden of anxiety and QoL are limited but not negligible, mainly due to overdiagnosis and treatment related issues.[83-90] Weighing the balance between the benefits and harms of MGUS screening is essential for decision making regarding screening at both the individual and the policy level.

Wilson and Jungner attempted to define screening criteria to guide the selection of conditions that would be suitable for screening.[91] These need to be carefully addressed when discussing screening programs and are listed below, with comments regarding MGUS:

1. The condition sought should be an important health problem.

The impact of MGUS, MM, lymphoproliferative diseases and amyloidosis is a very important health problem.

2. There should be an accepted treatment for patients with recognized disease.

Currently there are several accepted and highly effective treatment options for MM, lymphoproliferative diseases and amyloidosis.

3. Facilities for diagnosis and treatment should be available.

These are available for MGUS and related diseases.

4. There should be a recognizable latent or early symptomatic stage.

MGUS and smoldering MM are known and well-defined precursor states.

5. There should be a suitable test or examination.

Electrophoresis and FLC analyses are easy blood test to perform.

6. The test should be acceptable to the population.

A simple blood test is acceptable.

7. The natural history of the condition, including development from latent to declared disease, should be adequately understood.

This is known in MGUS.

8. There should be an agreed policy on whom to treat as patients.

There is an agreed policy, however this is a moving target and the trend is to treat earlier stages of disease.

9. The cost of case-finding (including diagnosis and treatment of patients diagnosed) should be economically balanced in relation to possible expenditure on medical care as a whole.

The cost for electrophoresis and FLC is not high, and cost can be kept low by including and collaborating with other national screening programs.

10. Case-finding should be a continuing process and not a "once and for all" project.

This is true for MGUS.

It is clear, as summarized above, that MGUS is a very common precursor conditions and most studies on work-up, follow-up, and monitoring are based on retrospective case series. As suggested by the Dr. Robert Kyle, who originally termed the condition and is the pioneer in the work of monoclonal gammopathies together with Dr Vincent Rajkumar: "*We need prospective studies to address the value of follow-up in MGUS and the optimal approach to such follow-up. The risk-adapted approach is a compromise that ensures that scarce resources are focused on the patients most likely to benefit. We also need studies to address the question of the possible merits of screening for the presence of MGUS in a normal, older population*."[71]

2. OBJECTIVES

The overall objective of this study is to evaluate the impact of screening for MGUS on overall survival and mortality in MM, lymphoproliferative diseases, and amyloidosis, and establish the optimal follow-up strategy. We propose to invite all individuals born 1975 or earlier in Iceland (approximately 140,000 individuals) to participate in a nationwide screening study for MGUS and smoldering MM and invite those that are diagnosed with MGUS and LC-MGUS to be included in a RCT to evaluate the impact of screening and the optimal follow-up strategy. Also we plan to use an innovative approach by improving risk models for progression. Hopefully our study will lead to a theoretical change in how risk models are conducted with inclusion of genetic, imaging, environmental, and biological factors. Also the impact of early diagnosis and treatment of smoldering MM and MM in a whole population will be evaluated.

2.1. Specific aims

1. To evaluate whether overall survival is improved by screening for MGUS.

2. To evaluate whether cause-specific survival is improved in screened MGUS individuals.

3. To evaluate the effect of screening for MGUS on quality of life, depression, and anxiety.

4. To evaluate the cost-effectiveness of screening for MGUS.

5. To evaluate the optimal follow-up strategy for individuals with MGUS-based on risk adapted criteria.

6. To evaluate the impact of screening for smoldering MM in a population.

7. To evaluate the effect of early treatment in a screened population of MM, lymphoproliferative diseases, and amyloidosis in a whole nation.

8. To identify the optimal approach to radiological and bone marrow examinations in MGUS.

9. To identify novel genetic variants as risk factors for development and evaluate the impact of germline variants on progression

10. To integrate clinical factors, protein markers (including immunoglobulin free light-chains) and immunophenotyping by flow cytometry with molecular profiles (including gene expression profiling), to predict disease progression.

Various exploratory analyses will also be performed to better understand the clinical picture, pathophysiological nature, and natural history of MGUS.

3. METHODOLOGY

3.1. Study design

In short the study design is to screen all participants with SPEP and FLC. Participants that have a positive screening will be randomized to three arms, of which two arms will be called into a clinical study center for two separate types of follow-up. Upon diagnosis of progression from MGUS, participants will be followed even closer and referred to Landspítali University Hospital for treatment as indicated. Participants that already have highly abnormal testing at baseline will not be randomized to these three arms but will all enter close follow up and referred to Landspítali University Hospital as indicated. Participant with normal screening testing will be asked to answer QoL questionnaires annually.

3.2. Study population

We will invite all individuals living in Iceland that are born 1975 and earlier to participate in the screening part of the study. From the Register Iceland (Þjóðskrá Íslands) we will obtain a complete list of these individuals, including full name, unique IDs and home address. Currently, more than of 140,000 individuals living in Iceland are born 1975 an earlier. All these individuals will be invited to participate by a letter sent to their home address. This letter will contain a brochure with information on MGUS, the study design, participation, and rights. Also it will include two ways of informed consent:

1. A letter with information of rights and duties of participants, which can be signed and posted via mail (free of charge for participants-cost covered by the project). This will be sent to the study coordinator and registered in an electronic database.

2. Electronic informed consent. Participants are also given the opportunity to log on to the study´s homepage: www.MGUS.is and log on with their identifier and a unique password for each participant or to use the government maintained e-certificate (see https://www.island.is/en/icekey-e---certificate/about-icekey/ for details on e-certificates). The participant will then get the opportunity to confirm consent or "I wish not to participate in the research".

3.2.1. Computerized database

A database with each individual, including name, ID, and home address will be built. Individuals who agree to participate will be registered in the electronic database including the ID. Individuals that have not refused participation will be contacted by a letter or a telephone call/text message. Individuals who do not want to participate will not be registered or contacted further.

3.2.2. Pilot Project

A pilot project to test the information material, the electronic informed consent, stress tests for the homepage, and recruitment strategies will be done. This will only include people from Akranes, a town with approximately 3,000 individuals 40 years or older, and will be performed a few weeks before the launch of the study.

3.2.3. Registries

The nationwide Icelandic *Cancer Registry*, which was established in 1951 has more than 99% completeness and over 96% diagnostic accuracy.[92] Information on prior and subsequent MM, lymphoproliferative diseases and other malignancies as well as prior MGUS will be obtained by cross linking with participant´s ID list.

The Icelandic *Patient Registry*, captures ICD codes throughout Iceland, including all in- and outpatient care.

Register Iceland includes a complete list of all residents of Iceland with dates of birth, dates of death (updated weekly).

Cause of Death Registry, digitally registered since 1971, based on death certificates and coded according to ICD-10 since 1996. It is a nationwide register that registers all deaths with primary cause of death and all underlying causes for all residents of Iceland.

The Icelandic Pharmaceutical Database, captures all electronically dispensed prescription medications and various attributes of the prescription (formulation, strength, amount etc.) since January 1st 2003 and mechanical dose dispensed prescription drugs since January 1st 2006. These do not include medications in the inpatient setting.

3.2.4. Specific risk groups

Information on family history will be included in the study project and family tree information obtained from genealogy database at Landspitali University Hospital. This will enable stratified analyses on screening particular risk groups, as MGUS and MM have been shown to aggregate within families.[19, 24] Also autoimmune disease has been described as a risk factor for MGUS and MM[35], and information on autoimmunity and other diseases, will be obtained from the Patient Registry. Iceland is predominantly a Caucasian population and thus we cannot compare the impact of screening among different races, which is a limitation, as black race is a known risk factor for MGUS and MM.[10, 25, 93] Overall, the inclusion of specific risk groups is feasible due to the unique infrastructure in Iceland with cross-linkage of personal IDs to different official registries with almost 100% completeness. This will allow us to perform a state of the art scientific approach that is not possible in other countries due to large number, lack of infrastructure, and high cost.

3.3. Protocol design

All participants will have two separate attributes, a group and a track. Groups are assigned after screening and indicate the finding of the screening study. A participant never exits their group. Tracks however define what follow-up and

testing is performed according to the protocol. Participants can change between tracks throughout the study. While groups do not directly direct the follow-up of participants, groups both constrain and affect what tracks participants are assigned.

These two attributes will guide each participant through the study and are illustrated in the overview flowchart.

3.3.1. Clinical team meeting

All findings, divergence from protocol, or other events considered relevant will be discussed in regularly scheduled clinical team meetings. These meetings will include clinical staff and the primary investigator. Each case will be reviewed with regard to the management of participants within their respective tracks as well as decisions on a change of track in participants where that may be indicated. Decisions will be made upon predetermined criteria (i.e. IMWG criteria) as well as at the discretion of the investigator.

3.3.2. Clinical study center

Located at Skógarhlíð 8, 105 Reykjavík, Iceland, the study center will be staffed by personnel that have received training on good clinical practice (GCP).

3.3.3. Groups

After screening testing has been performed participants will be assigned a group. Before screening, participants will be classified as being ungrouped. These groups are:

1. Abnormal non-MGUS group
2. MGUS group
3. Normal group

The groups will be further detailed under sections 3.5, 3.6, and 3.7.

3.3.4. Tracks

From the time of entering the study and exiting the study all participants will be assigned a specific track. Throughout the study, participants may change tracks multiple times. There is no limit on the number of track changes but each track has defined events in which a participant will change track. The tracks are:

3.3.4.1. Passive track (Track 0)

All participants enter the passive track after providing their informed consent to participating in the study. Participants in the non-MGUS group as well as some of the participants in the MGUS group will be assigned to this track as well. The track comprises of an annual quality of life questionnaire as well as screening. See track 0 flowchart.

3.3.4.2. Low risk track (Track 1)

Participants with low risk MGUS enter the low-risk track after randomization. The track follows IMWG guidelines for low risk MGUS and does not include a BM biopsy or imaging studies unless predetermined critera are met. See schedule of procedures arm 2-1 and track 1 low risk flowchart.

3.3.4.3. Light-chain MGUS, intermediate/high risk track (Track 2)

Participants with intermediate or high-risk MGUS in arm 2 of the MGUS group enter the intermediate/high risk track after randomization. The track follows IMWG guidelines for intermediate and high risk MGUS and routinely includes BM biopsy and imaging studies. For participants with light-chain MGUS is the same procedure excluding BM biopsy and imaging studies. See schedule of procedures arm 2-2, schedule of procedures arm 2-2 light chain and track 2 flowchart.

3.3.4.4. Arm3 track (Track 3)

All participants in arm 3 of the MGUS group enter the arm3 track. The track includes more intensive baseline testing and follow-up than arm2 tracks. The track also includes a lower threshold for further testing and changing to more intensive follow-up. See schedule of procedures arm 3 and track 3 flowchart.

3.3.4.5. Intensive4 track (Track 4)

All participants in the abnormal non-MGUS group will enter the intensive4 track. Participants in track 1, 2 and 3 change to the intensive4 track at progression or when closer follow-up is indicated. The track includes an extensive baseline visit and follow up every 4 months. Participants can be referred from this track to a hematologist for further work-up and treatment. See schedule of procedures for intensive4 track direct, arm 2, arm 3 and intensive4 track flowchart.

3.3.4.6. Intensive6 track (Track 5)

Participants on the intensive4 track that have been stable for twelve months will move to the intensive6 track. The visits in the intensive6 track are identical to those of the intensive4 track but occur every 6 months instead of every 4 months. Participants can be referred from this track to a hematologist for further work-up and treatment. See schedule of procedures for intensive6 track and intensive6 track flowchart.

3.3.4.7. Control track (Track 6)

A subset of participants in the non-MGUS group will be assigned to the control track. This includes a single visit identical to the first visit in track 3 except for BM biopsy and imaging. Participants then re-enter track 0. See the track 6 flowchart.

3.4. Screening

3.4.1. Blood sampling

For the first 3 years of the study, participants will not be contacted for blood sampling. Instead we will take advantage of the fact that most people that are 40 years or older, undergo blood sampling for various reasons during this 3 year period. We will cross-link the ID list from all participants in the computerized database to the database of all major laboratories in Iceland, which covers approximately 90% of all blood sampling in Iceland, including the Reykjavik area and surrounding towns, Akureyri, Akranes, Ísafjörður, Reykjanesbær and Selfoss. Software will be designed to link these two sources (1. consenting participants and 2. people undergoing blood sampling at different health care providers) so

that participants in the study will be flagged and samples for the study obtained. When blood is drawn from a participant in any of the above-mentioned centers for any reason, the software will flag the sample. At various other healthcare providers and clinical laboratories around the country, samples will be cross linked to consenting participants manually using the above-mentioned software. These samples will then be sent to a centralized lab where a 1-2 mL serum aliquot will be put in to a separate tube belonging to the study, which will be marked with an encrypted bar code unique for this individual. The samples serum will then be stored at 4°C. At regular individuals, a package including approximately 200 samples, anonymously marked with a unique barcode, without any identification, will be shipped to Binding site, in Birmingham UK, for analyses (see below). To prevent repeated measurements from the same individual, the database will be updated when an individual has already donated blood for the study.

3.4.2. Electrophoresis and free light chain analyses

SPEP, immunofixation, FLC, and Hevylite analyses from all participants will be performed at Binding Site laboratory that will fund the analyses (see Letter of Support). The Binding Site Group Ltd (TBS) is a biotechnology company that is committed to the research, development, manufacture and distribution of innovative immunodiagnostic assays for the global laboratory market. TBS has state-of-the art and fully equipped laboratories for assay development, production and validation. For these analyses 1-2 mL serum from each participant will be shipped from Iceland to the UK. We will evaluate the following:

a) The presence or absence of an M-protein
b) The concentration of the M-protein in g/L
c) The Isotype of the M-protein (IgG, IgA, or IgM)
d) The presence or absence of an abnormal kappa:lambda ratio
e) The kappa:lambda ratio and intact immunoglobulin monoclonal proteins.

Once the analyses have been performed, the results from these analyses will be sent to the study coordinator in Iceland.

3.5. Abnormal non-MGUS group

Participants with an M-protein level >30 g/L or an FLC ratio >100 are not considered to have MGUS. They have either SMM, MM, or WM. Therefore these individuals will be defined as a separate group after screening. They will not be randomized to different follow-up but will all enter track 4 (intensive4 track) at baseline. These participants are constrained to only follow track 4 or 5 (intensive4 and -6 respectively). They may switch between these two tracks as detailed in flowcharts 4 and 5.

3.6. MGUS group

Participants found to have MGUS on screening will enter a randomized controlled trial and randomized to 3 separate arms.

3.6.1. Inclusion criteria:

1. Written or electronic informed consent to participation in the iStopMM trial.
2. M-component present on electrophoreses or abnormal FLC ratio

3.6.2. Exclusion criteria:

1. Prior MM, lymphoproliferative disease, or amyloidosis
2. M-protein ≥30 g/L
3. FLC ratio ≥100

3.6.3. Arm 1

Participants will receive routine care outside the bounds of the trial. No further work-up. Participants will be blinded to the presence of MGUS. All participants in arm 1 will be assigned to track 0. If an interim analysis shows a poorer outcome for arm 1 as compared to arm 2 and 3, participants in arm 1 will be unblinded and randomized to arm 2 and 3 in the same manner as described below.

3.6.4. Arm 2

Following International Myeloma Working Group (IMWG) guidelines participants will be classified as having low, intermediate, or high risk MGUS (see table 1).

Criteria	Classification
• IgG M-protein<br>• M-protein <15 g/L<br>• Normal FLC ratio	• Low risk: 3<br>• Intermediate risk: 1-2<br>• High risk: 0

Table 1: The Mayo risk criteria for MGUS. These criteria will be used to classify participants in arm two according to risk.

Low risk participants will enter track 1 (low risk track) and may change to track 2 (intermediate/high risk track) or track 4 (intensive4 track) as detailed in the track 1 flowchart. Participants will not re-enter track 1 after entering track 2 but may stay on track 1 if they need an additional visit and there are no pathological findings after the additional visit.

Intermediate/high risk participants will enter track 2 (intermediate/high risktrack) and may change to track 4 as detailed in the track 2 flowchart. Participants may re-enter track 2 from track 4 or 5 if close follow-up is no longer indicated.

3.6.5. Arm 3

Participants in arm 3 will receive more detailed baseline work-up as well as more intensive follow-up with a lower threshold for repeat. All participants will receive the same follow-up regardless of their risk group.

All participants will enter track 3 (arm3 track) and may change to track 4 (intensive4 track) as detailed in the track 3 flowchart. Participants may re-enter track 3 from track 4 if close follow-up is no longer indicated. Participants in arm 3 that move from track 4 to track 5 (intensive6) track may change back to track 3 if indicated.

3.6.6. Randomization and blinding

Participants will be randomized in the following blocks across arms 1,2, and 3:

- M-component < 15 g/L
- M-component ≥15 g/L
- LC-MGUS with kappa level ≥38.93 g/L or lambda level ≥35.85 g/L
- LC-MGUS with kappa level <38.93 g/L or lambda level <35.85 g/L

Participants are randomized in a non-static and non-pre-ordered manner as they are included in the study. This randomization will take into account how participants have previously been randomized to the three arms at the time of randomization. Participants with a previous diagnosis of MGUS outside the bounds of the trial will only be randomized to arm 2 or 3 in the same manner.

Investigators will be blinded to what participants are in arm 1. Their encrypted study number will not be decoded while participants are still in arm 1.

3.7. Normal group

Participants with normal findings on screening SPEP and FLC will enter the normal group. They will all continue on track 0 (passive track), receiving regular quality of life questionnaires.

3.7.1. Controls

A sub-group of the normal group will act as controls. They will be matched to participants in arm 2 and 3 by age and gender. They will enter track 6 (control track) and then re-enter track 5 (passive track).

3.7.2. Blinding

Investigators will be blinded to what participants are in the normal group. Their encrypted study number will not be decoded unless called in as a control.

3.8. Onset of new symptoms

All participants arriving to the clinical study center will be asked to contact the clinical study center if new symptoms or signs that might indicate progression should arise. Participants will be provided with a flyer detailing these symptoms. The flyer for participants with IgG, IgA and light chain MGUS includes:

- Hospitalization
- Diagnosis of enlarged lymph node
- Fracture
- New pain lasting at least a week
- Worsening fatigue lasting at least a week
- Diagnosis of anemia, renal failure, hyepercalcemia
- Participants with IgM MGUS will get a different flyer that includes:Hospitalization
- Diagnosis of enlarged lymph node, liver or spleen
- Unexplained weightloss
- Fever without infection
- Repeated night sweats

- Worsening fatigue lasting at least a week
- Diagnosis of anemia

These symptoms will be recorded and brought up in the next clinical meeting. Should re-evaluation be indicated participants might be moved to track 4 (intensive4 track) and called in for a visit at the discretion of the investigator.

3.9. Outcome measures in RCT

All participants will be followed for progression to MM, lymphoproliferative disorders as well as other cancers, by cross linking to the Cancer Registry and for other diseases by cross-linking to the Patient Registry. Information on all subsequent cancer diagnosis and other diseases (based on ICD-codes), both prior and subsequent to inclusion and information on vital status will be gathered with almost 100% accuracy.

The study arms will also be cross-linked to the Patient Register to obtain information other sub-sequent non-malignant disease.

Data on vital status will be obtained at regular intervals (every three months) and obtained from Registers Iceland. Information on survival in selected cancers will be obtained by using date of cancer diagnosis and date of death, or date at the time of analyses.

3.10. Medical history and physical examination

Medical history and baseline information will be collected at the first visit using questionnaires approved by the scientific ethics committee (See appendix). Some but not all of these questionnaires will be answered repeatedly over the study period while others only at baseline.

Information on prescription drug use, malignant and non-malignant disease, and hospitalization will be retrieved from the registries specified in section 3.2.3.

Careful relevant physical examination will be performed repeatedly at the clinical study center.

3.11. Assessments of quality of life, anxiety and depression

To evaluate whether knowledge of MGUS is associated with anxiety, depression, or a decreased QoL participants will answer questionnaires (See appendix) approved by the scientific ethics committee. These questionnaires will be answered electronically on the study website (www.MGUS.is) at time of informed consent, thus providing baseline results before a diagnosis of MGUS has been made or not. Subsequently, all participants in the study will be asked to answer the same questionnaires annually. Participants called into the clinical study center will be asked to answer the questionnaires upon arrival at the clinical study center.

By using this approach, a good estimate of QoL changes can be detected, both by internal comparison, i.e. QoL results compared in persons before and after they received the diagnosis, and by comparing changes over time in MGUS versus others.

3.12. Assessment of cost-effectiveness

Calculations of cost-effectiveness will be performed in collaboration with collaboration with the Department of economics at the University of Iceland and will include standard estimates as deemed appropriate.

3.13. Assessment of cardiac function

Track 3, tracks 4 (intensive4) and track 5 (intensive6) excluding participants from arm 2 that are on intensive track) include performing a 12-lead electrocardiogram (ECG) and measurements of the cardiac markers pro-BNP and TnT to assess cardiac function. Abnormalities in pro-BNP or TnT not explained by concurrent renal impairment will trigger referral of the participant to a cardiologist for further evaluation, including cardiac echocardiography.

3.14. Assessment of peripheral neuropathy

All participants will answer questionnaires regarding symptoms of peripheral neuropathy and neuropathic pain (see appendix). All participants that arrive at the clinic may also, alongside physical examination, undergo a nerve conduction study of the sural nerve using the hand held, bed-side, DPN-check device by Neurometrix. This is to screen participants for peripheral neuropathy.

Participants with abnormal findings may be referred on to a neurologist, an endocrinologist, or to a hematologist for further work-up or treatment as deemed appropriate.

3.15. Assessment for hepatomegaly

Liver size will be determined in every physical examination performed and on all CT scans performed. If hepatomegaly is detected serum or plasma measurements of alanine aminotransferase (ALAT), aspartate aminotransferase (ASAT), alkaline phosphatase (ALP), and bilirubin will be measured and the participants in question referred to the gastroenterology department at Landspítali University Hospital.

3.16. Germline genetics

Studies on germline genetics will be performed in collaboration with DeCODE genetics. All participants arriving at the clinical study center will be asked for their separate informed consent for participating in such studies. Genetic information already gathered by DeCODE genetics may be used for analysing this data.

3.17. Flow cytometry

A sub-study of participants in arm 3 and controls will include flow cytometry of BM and peripheral blood. Flow cytometry results will not affect the course of follow-up within the iStopMM trial.

3.17.1 Study population

All participation in the flow cytometry sub-study will come from arm 3 of the MGUS group. They will be selected on an "as-they-come" basis, i.e. as many participants as the flow cytometry lab can handle will be selected from the arm 3

participants arriving for a baseline examination to the clinical center each day. All participants with an M-component >15 g/L will be included and if the lab has residual capacity other participants will be randomized to fill that capacity. Once selected into the flow cytometry sub-study participants will continue to provide samples into said sub-study. Controls from the normal group will also be included in the flow cytometery sub-study.

3.17.2. Samples studied by flow cytometry

For participants coming from arm 3, BM and peripheral blood will be analyzed by flow cytometry at baseline. Peripheral blood collected repeatedly at follow-up visits will also be analyzed. If BM aspirate and biopsy is required during the study period or at the end of the study that sample will also be analyzed by flow cytometry.

For a subset of the controls, peripheral blood collected at the control visit will be analyzed by flow cytometry.

3.17.3. Protocols and panels

Flow cytometry will be performed in accordance with protocols from the EuroFlow consortium. They have previously been optimized by flow laboratories across Europe. The EuroFlow panel previously designed to determine minimal residual disease in MM (MM MRD) will be used on BM and peripheral blood samples. Other panels may be added to further study the environment of the myeloma cells.

3.18. Biobanking

Samples will be collected from participants and stored in a biobank at a facility operated by DeCODE genetics. Participants will provide a separate informed consent for donating samples to the biobank. Samples of BM, peripheral blood, and urine will be collected at different frequency and at different time points. These time points are detailed in schedules of procedures and flowcharts 2,3,4,5, and 6. The samples will be taken out of the biobank as deemed appropriate, pending the approval of the scientific ethics committee.

3.18.1 Bone marrow

All BM samples will be stored. This will include biopsies and one aspirated fraction that will be fixed and paraffin embedded. Aspirate will further be separated into BM supernatant, CD138+ cells, and CD138- cells. These samples will be aliquoted and biobanked.

3.18.2. Peripheral blood

At the same time as BM is biobanked as well as at pre-determined time points, serum, plasma, cell free plasma (cfPlasma) as well as whole blood in EDTA- and PAXgene tubes will also be aliquoted biobanked.

3.18.3. Urine

Urine will also be collected pre-determined time points. Samples will be aliquoted and biobanked.

4. MONITORING

4.1. Interim analyses

The first interim analysis will be performed after 500 subjects have been followed for at least 6 months. This is anticipated to be approximately 7-8 months after first subject has been randomized. The purpose of this interim analysis is to have a comprehensive evaluation of safety, because there are limited safety data for screening for MGUS. The second interim analysis will be performed when 100 events (deaths) have occurred. This is anticipated to be approximately 11-12 months after the first subject has been randomized. The purpose of this interim analysis is to evaluate cumulative interim safety and efficacy data. In addition to the above-described interim analyses, biannual interim analyses will be performed for safety, and to evaluate of the primary objective of the study has been reached. The DMC will carefully review the interim results for recommendation of early termination due to overwhelming efficacy or lack of efficacy.

In case arm 1 has worse outcome, individuals in arm 1 will be unblinded. These individuals will be invited for a medical appointment and informed about the findings. They will then be invited to continue participation in the trial and consequently randomized to the remaining 2 arms of the study. This is to evaluate the most optimal follow-up strategy for MGUS. Those refusing further participation will be referred to a hematologist for further work-up and follow-up. In case the study objectives are reached, the RCT part will be terminated and individuals informed about their MGUS. In case arm 2 or 3 have inferior outcome the study will be subject to evaluation by the DMC.

4.2. Data monitoring committee

An independent data monitoring committee (DMC) will be established for the RCT part of the study, for evaluating risk/benefit when possible and evaluating the efficacy of the treatment at the time of interim analysis in study. The primary purpose of this DMC is to ensure the safety of the subjects in this study by monitoring safety data collected in the clinical program and to evaluate the interim safety and efficacy data and to provide recommendations to the study team. The DMC will make recommendations concerning the conduct of the study. The DMC will be comprised of two clinicians and one statistician.

4.3.Clinical monitoring

Clinical monitoring will be done by an independent and experienced study monitor. The study monitor will monitor the study conduct at the clinical study center for compliance with applicable GCP principles.

5. DATA MANAGEMENT

5.1. Data management system

The data management system used to register information from each individual from the RCT, including data from medical history, examination, and laboratory

work will be designed using REDCap. REDCap (Research Electronic Data Capture) is a secure, web-based application for building and managing online research surveys and databases. REDCap is a free resource available to researchers at the University of Iceland and their external team members. One of the investigators (ÞJL) has led the implementation of REDCap at the University of Iceland and Landspitali University Hospital. Source data collected at the study site will be recorded directly into REDCap.

5.2. Data storage

Data from the TBS, which includes only non-traceable codes, will be sent through the secure Syncplicity data-sharing site. Only researcher in the study will have access to the data and data will never be transferred to another institution. All other data will be kept on servers operated by the University of Iceland in a secure data environment.

5.3. Identifier encryption

The study database will be linked to other databases using a one-way encrypted national identification number. National identifiers will be encrypted using a one-way algorithm, but with a secret code added to reduce the risk of a successful list-based brute force attack. The secret code will be destroyed after the end of the study when the data have been stored in a research data repository (safn heilbrigðisupplýsinga). Once the secret code has been destroyed there will be no method of linking the study database to individuals again.

6. STATISTICAL ANALYSIS

Prevalence of MGUS and LC-MGUS, with 95% confidence intervals (CIs), will be estimated with a binomial test. Analysis of numerical outcomes will be done with a multivariate linear regression model, whereas analysis of binary outcomes will be done with a multivariate logistic regression or linear poisson regression as deemed appropriate. Overall, as well as cause-specific, survival from the day of randomization, with 95% CIs, will be estimated with the Kaplan-Meier estimator. Comparison of survival between study groups will be done with a Cox proportional hazard regression and hazard ratios (HR) reported with 95% CIs. Several sub-analysis with stratification by gender, age-group and other relevant categories as appropriate will be performed. Alpha level of 0.05 will be used in all analyses. Additional statistical may be added for exploratory analyses.

7. ETHICS

One of the main ethical issues is the fact that individuals randomized to Arm 1 of the study will not get information on their MGUS and will undergo routine clinical care and thus not be worked up or followed beyond that what normally happens in the population. This is in our mind ethically defendable for several reasons.

First, approval for this study will be obtained from the Ethical Committee in Iceland.

Second, we already know that 4.2% of the population over the age of 50 years have MGUS and these are not being captured or evaluated.

Third, we will not randomize individuals with M-protein>30 g/L or involved:uninvolved serum FLC ratio ≥100 in order not to miss underlying MM.

Fourth, we have pre-planned several interim analyses to detect any differences in outcome and will terminate the study and unblind individuals in Arm 1 in case of inferior efficacy (after recommendation from independent DCM).

Fifth, informed consent will be obtained from all participants. They will be thoroughly informed about the study design and can whenever they want decide to stop participation.

Sixth, individuals in the RCT will still be able and encouraged to seek medical help in cases of sign/symptoms of any serious illness and can be diagnosed with MM, MGUS or any other disorder as other people in the general population.

Another ethical issue is the potential harm of screening. Screening is by no means uncontroversial as knowledge of a medical condition may lead to unnecessary stress and testing. As with all clinical trials this trial has some risks but we think the current literature clearly indicates the potential benefits outweigh the potential harm. It is still of great importance to study the potential harms of screening and by assessing QoL at regular intervals and by interim analysis we will be able to closely monitor the potential harm.

This protocol and informed consent have the approval of The National Bioethics Committee and The Icelandic Data Protection Authority. All changes made to the protocol or informed consent will be submitted to the Ethics Committee for approval. All recruitment procedures, documentation and information to participants has been approved by the Ethical committee. All information about participants is confidential. This study will be conducted in adherence with the Helsinki declaration. Investigators ascertain they will apply due diligence to avoid protocol deviations. The study will be conducted in compliance with applicable GCP principles and the staff trained accordingly.

8. REFERENCES

1. Rajkumar SV, Dimopoulos MA, Palumbo A, Blade J, Merlini G, Mateos MV, Kumar S, Hillengass J, Kastritis E, Richardson P, Landgren O, Paiva B, Dispenzieri A, Weiss B, LeLeu X, Zweegman S, Lonial S, Rosinol L, Zamagni E, Jagannath S, Sezer O, Kristinsson SY, Caers J, Usmani SZ, Lahuerta JJ, Johnsen HE, Beksac M, Cavo M, Goldschmidt H, Terpos E, Kyle RA, Anderson KC, Durie BG, Miguel JF. International Myeloma Working Group updated criteria for the diagnosis of multiple myeloma. *The lancet oncology* 2014; **15**(12): e538-48.

2. Kyle RA. Monoclonal gammopathy of undetermined significance. Natural history in 241 cases. *The American journal of medicine* 1978; **64**(5): 814-26.

3. Socialstyrelsen. Cancer Incidence in Sweden 2006. Stockholm, Sweden; 2007.

4. Kristinsson SY, Landgren O, Dickman PW, Derolf AR, Bjorkholm M. Patterns of survival in multiple myeloma: a population-based study of patients diagnosed in Sweden from 1973 to 2003. *Journal of clinical oncology : official journal of the American Society of Clinical Oncology* 2007; **25**(15): 1993-9.

5. Criteria for the classification of monoclonal gammopathies, multiple myeloma and related disorders: a report of the International Myeloma Working Group. *Br J Haematol* 2003; **121**(5): 749-57.

6. Gay F, Larocca A, Wijermans P, Cavallo F, Rossi D, Schaafsma R, Genuardi M, Romano A, Liberati AM, Siniscalchi A, Petrucci MT, Nozzoli C, Patriarca F, Offidani M, Ria R, Omede P, Bruno B, Passera R, Musto P, Boccadoro M, Sonneveld P, Palumbo A. Complete response correlates with long-term progression-free and overall survival in elderly myeloma treated with novel agents: analysis of 1175 patients. *Blood* 2011; **117**(11): 3025-31.

7. Turesson I, Velez R, Kristinsson SY, Landgren O. Patterns of improved survival in patients with multiple myeloma in the twenty-first century: a population-based study. *Journal of clinical oncology : official journal of the American Society of Clinical Oncology* 2010; **28**(5): 830-4.

8. Brenner H, Gondos A, Pulte D. Recent major improvement in long-term survival of younger patients with multiple myeloma. *Blood* 2008; **111**(5): 2521-6.

9. Kumar SK, Rajkumar SV, Dispenzieri A, Lacy MQ, Hayman SR, Buadi FK, Zeldenrust SR, Dingli D, Russell SJ, Lust JA, Greipp PR, Kyle RA, Gertz MA. Improved survival in multiple myeloma and the impact of novel therapies. *Blood* 2008; **111**(5): 2516-20.

10. Waxman AJ, Mink PJ, Devesa SS, Anderson WF, Weiss BM, Kristinsson SY, McGlynn KA, Landgren O. Racial disparities in incidence and outcome in multiple myeloma: a population-based study. *Blood* 2010; **116**(25): 5501-6.

11. Kumar SK, Dispenzieri A, Lacy MQ, Gertz MA, Buadi FK, Pandey S, Kapoor P, Dingli D, Hayman SR, Leung N, Lust J, McCurdy A, Russell SJ, Zeldenrust SR, Kyle RA, Rajkumar SV. Continued improvement in survival in multiple myeloma: changes in early mortality and outcomes in older patients. *Leukemia* 2014; **28**(5): 1122-8.

12. Brenner H, Gondos A, Pulte D. Expected long-term survival of patients diagnosed with multiple myeloma in 2006-2010. *Haematologica* 2009; **94**(2): 270-5.

13. Dispenzieri A, Katzmann JA, Kyle RA, Larson DR, Melton LJ, 3rd, Colby CL, Therneau TM, Clark R, Kumar SK, Bradwell A, Fonseca R, Jelinek DF, Rajkumar SV. Prevalence and risk of progression of light-chain monoclonal gammopathy of undetermined significance: a retrospective population-based cohort study. *Lancet* 2010; **375**(9727): 1721-8.

14. Rajkumar SV, Kyle RA, Buadi FK. Advances in the diagnosis, classification, risk stratification, and management of monoclonal gammopathy of undetermined significance: implications for recategorizing disease entities in the presence of evolving scientific evidence. *Mayo Clinic proceedings Mayo Clinic* 2010; **85**(10): 945-8.

15. Kyle RA, Therneau TM, Rajkumar SV, Larson DR, Plevak MF, Offord JR, Dispenzieri A, Katzmann JA, Melton LJ, 3rd. Prevalence of monoclonal gammopathy of undetermined significance. *The New England journal of medicine* 2006; **354**(13): 1362-9.

16. Axelsson U, Bachmann R, Hallen J. Frequency of pathological proteins (M-components) om 6,995 sera from an adult population. *Acta Med Scand* 1966; **179**(2): 235-47.

17. Harris TB, Launer LJ, Eiriksdottir G, Kjartansson O, Jonsson PV, Sigurdsson G, Thorgeirsson G, Aspelund T, Garcia ME, Cotch MF, Hoffman HJ, Gudnason V. Age, Gene/Environment Susceptibility-Reykjavik Study: multidisciplinary applied phenomics. *American journal of epidemiology* 2007; **165**(9): 1076-87.

18. Kristinsson SY, Holmberg E, Blimark C. Treatment for high-risk smoldering myeloma. *The New England journal of medicine* 2013; **369**(18): 1762-3.

19. Kristinsson SY, Bjorkholm M, Goldin LR, Blimark C, Mellqvist UH, Wahlin A, Turesson I, Landgren O. Patterns of hematologic malignancies and solid tumors among 37,838 first-degree relatives of 13,896 patients with multiple myeloma in Sweden. *Int J Cancer* 2009; **125**(9): 2147-50.

20. Kristinsson SY, Bjorkholm M, Goldin LR, McMaster ML, Turesson I, Landgren O. Risk of lymphoproliferative disorders among first-degree relatives of lymphoplasmacytic lymphoma/Waldenstrom macroglobulinemia patients: a population-based study in Sweden. *Blood* 2008; **112**(8): 3052-6.

21. Kristinsson SY, Goldin LR, Bjorkholm M, Koshiol J, Turesson I, Landgren O. Genetic and immune-related factors in the pathogenesis of lymphoproliferative and plasma cell malignancies. *Haematologica* 2009; **94**(11): 1581-9.

22. Kristinsson SY, Goldin LR, Bjorkholm M, Turesson I, Landgren O. Risk of solid tumors and myeloid hematological malignancies among first-degree relatives of patients with monoclonal gammopathy of undetermined significance. *Haematologica* 2009; **94**(8): 1179-81.

23. Kristinsson SY, Koshiol J, Goldin LR, Bjorkholm M, Turesson I, Gridley G, McMaster ML, Landgren O. Genetics- and immune-related factors in the pathogenesis of lymphoplasmacytic lymphoma/ Waldenstrom's macroglobulinemia. *Clin Lymphoma Myeloma* 2009; **9**(1): 23-6.

24. Landgren O, Kristinsson SY, Goldin LR, Caporaso NE, Blimark C, Mellqvist UH, Wahlin A, Bjorkholm M, Turesson I. Risk of plasma cell and lymphoproliferative disorders among 14621 first-degree relatives of 4458 patients with monoclonal gammopathy of undetermined significance in Sweden. *Blood* 2009; **114**(4): 791-5.

25. Landgren O, Gridley G, Turesson I, Caporaso NE, Goldin LR, Baris D, Fears TR, Hoover RN, Linet MS. Risk of monoclonal gammopathy of undetermined significance (MGUS) and subsequent multiple myeloma among African American and white veterans in the United States. *Blood* 2006; **107**(3): 904-6.

26. Landgren O, Katzmann JA, Hsing AW, Pfeiffer RM, Kyle RA, Yeboah ED, Biritwum RB, Tettey Y, Adjei AA, Larson DR, Dispenzieri A, Melton LJ, 3rd, Goldin LR, McMaster ML, Caporaso NE, Rajkumar SV. Prevalence of monoclonal gammopathy of undetermined significance among men in Ghana. *Mayo Clin Proc* 2007; **82**(12): 1468-73.

27. Chubb D, Weinhold N, Broderick P, Chen B, Johnson DC, Forsti A, Vijayakrishnan J, Migliorini G, Dobbins SE, Holroyd A, Hose D, Walker BA, Davies FE, Gregory WA, Jackson GH, Irving JA, Pratt G, Fegan C, Fenton JA, Neben K, Hoffmann P, Nothen MM, Muhleisen TW, Eisele L, Ross FM, Straka C, Einsele H, Langer C, Dorner E, Allan JM, Jauch A, Morgan GJ, Hemminki K, Houlston RS, Goldschmidt H. Common variation at 3q26.2, 6p21.33, 17p11.2 and 22q13.1 influences multiple myeloma risk. *Nature genetics* 2013; **45**(10): 1221-5.

28. Broderick P, Chubb D, Johnson DC, Weinhold N, Forsti A, Lloyd A, Olver B, Ma YP, Dobbins SE, Walker BA, Davies FE, Gregory WA, Child JA, Ross FM, Jackson GH, Neben K, Jauch A, Hoffmann P, Muhleisen TW, Nothen MM, Moebus S, Tomlinson IP, Goldschmidt H, Hemminki K, Morgan GJ, Houlston RS. Common variation at 3p22.1 and 7p15.3 influences multiple myeloma risk. *Nature genetics* 2012; **44**(1): 58-61.

29. Weinhold N, Johnson DC, Chubb D, Chen B, Forsti A, Hosking FJ, Broderick P, Ma YP, Dobbins SE, Hose D, Walker BA, Davies FE, Kaiser MF, Li NL, Gregory WA, Jackson GH, Witzens-Harig M, Neben K, Hoffmann P, Nothen MM, Muhleisen TW, Eisele L, Ross FM, Jauch A, Goldschmidt H, Houlston RS, Morgan GJ, Hemminki K. The CCND1 c.870G>A polymorphism is a risk factor for t(11;14)(q13;q32) multiple myeloma. *Nature genetics* 2013; **45**(5): 522-5.

30. Swaminathan B, Thorleifsson G, Joud M, Ali M, Johnsson E, Ajore R, Sulem P, Halvarsson BM, Eyjolfsson G, Haraldsdottir V, Hultman C, Ingelsson E, Kristinsson SY, Kahler AK, Lenhoff S, Masson G, Mellqvist UH, Mansson R, Nelander S, Olafsson I, Sigurethardottir O, Steingrimsdottir H, Vangsted A, Vogel U, Waage A, Nahi H, Gudbjartsson DF, Rafnar T, Turesson I, Gullberg U, Stefansson K, Hansson M, Thorsteinsdottir U, Nilsson B. Variants in ELL2 influencing immunoglobulin levels associate with multiple myeloma. *Nat Commun* 2015; **6**: 7213.

31. Kristinsson SY, Koshiol J, Bjorkholm M, Goldin LR, McMaster ML, Turesson I, Landgren O. Immune-related and inflammatory conditions and risk of lymphoplasmacytic lymphoma or Waldenstrom macroglobulinemia. *J Natl Cancer Inst* 2010; **102**(8): 557-67.

32. Landgren O, Linet MS, McMaster ML, Gridley G, Hemminki K, Goldin LR. Familial characteristics of autoimmune and hematologic disorders in 8,406 multiple myeloma patients: a population-based case-control study. *Int J Cancer* 2006; **118**(12): 3095-8.

33. Landgren O, Rapkin JS, Mellemkjaer L, Gridley G, Goldin LR, Engels EA. Respiratory tract infections in the pathway to multiple myeloma: a population-based study in Scandinavia. *Haematologica* 2006; **91**(12): 1697-700.

34. Gregersen H, Pedersen G, Svendsen N, Thulstrup AM, Sorensen HT, Schonheyder HC. Multiple myeloma following an episode of community-acquired pneumococcal bacteraemia or meningitis. *Apmis* 2001; **109**(11): 797-800.

35. Lindqvist EK, Goldin LR, Landgren O, Blimark C, Mellqvist UH, Turesson I, Wahlin A, Bjorkholm M, Kristinsson SY. Personal and family history of immune-related conditions increase the risk of plasma cell disorders: a population-based study. *Blood* 2011; **118**(24): 6284-91.

36. Kristinsson SY, Tang M, Pfeiffer RM, Bjorkholm M, Blimark C, Mellqvist UH, Wahlin A, Turesson I, Landgren O. Monoclonal gammopathy of undetermined significance and risk of skeletal fractures: a population-based study. *Blood* 2010; **116**(15): 2651-5.

37. Melton LJ, 3rd, Rajkumar SV, Khosla S, Achenbach SJ, Oberg AL, Kyle RA. Fracture risk in monoclonal gammopathy of undetermined significance. *J Bone Miner Res* 2004; **19**(1): 25-30.

38. Gregersen H, Jensen P, Gislum M, Jorgensen B, Sorensen HT, Norgaard M. Fracture risk in patients with monoclonal gammopathy of undetermined significance. *Br J Haematol* 2006; **135**(1): 62-7.

39. Bida JP, Kyle RA, Therneau TM, Melton LJ, 3rd, Plevak MF, Larson DR, Dispenzieri A, Katzmann JA, Rajkumar SV. Disease associations with monoclonal gammopathy of undetermined significance: a population-based study of 17,398 patients. *Mayo Clin Proc* 2009; **84**(8): 685-93.

40. Melton LJ, 3rd, Kyle RA, Achenbach SJ, Oberg AL, Rajkumar SV. Fracture risk with multiple myeloma: a population-based study. *J Bone Miner Res* 2005; **20**(3): 487-93.

41. Pepe J, Petrucci MT, Nofroni I, Fassino V, Diacinti D, Romagnoli E, Minisola S. Lumbar bone mineral density as the major factor determining increased prevalence of vertebral fractures in monoclonal gammopathy of undetermined significance. *British journal of haematology* 2006; **134**(5): 485-90.

42. Gregersen H, Madsen KM, Sorensen HT, Schonheyder HC, Ibsen JS, Dahlerup JF. The risk of bacteremia in patients with monoclonal gammopathy of undetermined significance. *Eur J Haematol* 1998; **61**(2): 140-4.

43. Kristinsson SY, Tang M, Pfeiffer RM, Bjorkholm M, Goldin LR, Blimark C, Mellqvist UH, Wahlin A, Turesson I, Landgren O. Monoclonal gammopathy of undetermined significance and risk of infections: a population-based study. *Haematologica* 2011.

44. Kristinsson SY, Fears TR, Gridley G, Turesson I, Mellqvist UH, Bjorkholm M, Landgren O. Deep vein thrombosis after monoclonal gammopathy of undetermined significance and multiple myeloma. *Blood* 2008; **112**(9): 3582-6.

45. Kristinsson SY, Pfeiffer R, Björkholm M, Goldin LR, Schulman S, Blimark C, Mellqvist UH, Wahlin A, Turesson I, Landgren O. Arterial and venous thrombosis in monoclonal gammopathy of undetermined significance and multiple myeloma: a population-based study. *Blood* 2010; **In press**.

46. Mailankody S, Pfeiffer RM, Kristinsson SY, Korde N, Bjorkholm M, Goldin LR, Turesson I, Landgren O. Risk of acute myeloid leukemia and myelodysplastic syndromes after multiple myeloma and its precursor disease (MGUS). *Blood* 2011; **118**(15): 4086-92.

47. Kyle RA, Therneau TM, Rajkumar SV, Offord JR, Larson DR, Plevak MF, Melton LJ, 3rd. A long-term study of prognosis in monoclonal gammopathy of undetermined significance. *The New England journal of medicine* 2002; **346**(8): 564-9.

48. Gregersen H, Ibsen J, Mellemkjoer L, Dahlerup J, Olsen J, Sorensen HT. Mortality and causes of death in patients with monoclonal gammopathy of undetermined significance. *Br J Haematol* 2001; **112**(2): 353-7.

49. Kyle RA, Therneau TM, Rajkumar SV, Larson DR, Plevak MF, Melton LJ, 3rd. Long-term follow-up of 241 patients with monoclonal gammopathy of undetermined significance: the original Mayo Clinic series 25 years later. *Mayo Clin Proc* 2004; **79**(7): 859-66.

50. Schaar CG, le Cessie S, Snijder S, Franck PF, Wijermans PW, Ong C, Kluin-Nelemans H. Long-term follow-up of a population based cohort with monoclonal proteinaemia. *Br J Haematol* 2008.

51. Kyle RA, Therneau TM, Rajkumar SV, Remstein ED, Offord JR, Larson DR, Plevak MF, Melton LJ, 3rd. Long-term follow-up of IgM monoclonal gammopathy of undetermined significance. *Blood* 2003; **102**(10): 3759-64.

52. Kristinsson SY, Bjorkholm M, Andersson TM, Eloranta S, Dickman PW, Goldin LR, Blimark C, Mellqvist UH, Wahlin A, Turesson I, Landgren O. Patterns of survival and causes of death following a diagnosis of monoclonal gammopathy of undetermined significance: a population-based study. *Haematologica* 2009; **94**(12): 1714-20.

53. Kyle RA, Remstein ED, Therneau TM, Dispenzieri A, Kurtin PJ, Hodnefield JM, Larson DR, Plevak MF, Jelinek DF, Fonseca R, Melton LJ, 3rd, Rajkumar SV. Clinical course and prognosis of smoldering (asymptomatic) multiple myeloma. *N Engl J Med* 2007; **356**(25): 2582-90.

54. Weiss BM, Abadie J, Verma P, Howard RS, Kuehl WM. A monoclonal gammopathy precedes multiple myeloma in most patients. *Blood* 2009; **113**(22): 5418-22.

55. Landgren O, Kyle RA, Pfeiffer RM, Katzmann JA, Caporaso NE, Hayes RB, Dispenzieri A, Kumar S, Clark RJ, Baris D, Hoover R, Rajkumar SV. Monoclonal gammopathy of undetermined significance (MGUS) consistently precedes multiple myeloma: a prospective study. *Blood* 2009; **113**(22): 5412-7.

56. Rajkumar SV, Kyle RA, Therneau TM, Melton LJ, Bradwell AR, Clark RJ, Larson DR, Plevak MF, Dispenzieri A, Katzmann JA. Serum free light chain ratio is an independent risk factor for progression in monoclonal gammopathy of undetermined significance. *Blood* 2005; **106**(3): 812-7.

57. Turesson I, Kovalchik SA, Pfeiffer RM, Kristinsson SY, Goldin LR, Drayson MT, Landgren O. Monoclonal gammopathy of undetermined significance and risk of lymphoid and myeloid malignancies: 728 cases followed up to 30 years in Sweden. *Blood* 2014; **123**(3): 338-45.

58. Perez-Persona E, Vidriales MB, Mateo G, Garcia-Sanz R, Mateos MV, de Coca AG, Galende J, Martin-Nunez G, Alonso JM, de Las Heras N, Hernandez JM, Martin A, Lopez-Berges C, Orfao A, San Miguel JF. New criteria to identify risk of

progression in monoclonal gammopathy of uncertain significance and smoldering multiple myeloma based on multiparameter flow cytometry analysis of bone marrow plasma cells. *Blood* 2007; **110**(7): 2586-92.

59. Cherry BM, Korde N, Kwok M, Manasanch EE, Bhutani M, Mulquin M, Zuchlinski D, Yancey MA, Maric I, Calvo KR, Braylan R, Stetler-Stevenson M, Yuan C, Tembhare P, Zingone A, Costello R, Roschewski MJ, Landgren O. Modeling progression risk for smoldering multiple myeloma: results from a prospective clinical study. *Leukemia & lymphoma* 2013; **54**(10): 2215-8.

60. Dimopoulos M, Terpos E, Comenzo RL, Tosi P, Beksac M, Sezer O, Siegel D, Lokhorst H, Kumar S, Rajkumar SV, Niesvizky R, Moulopoulos LA, Durie BG, Imwg. International myeloma working group consensus statement and guidelines regarding the current role of imaging techniques in the diagnosis and monitoring of multiple Myeloma. *Leukemia* 2009; **23**(9): 1545-56.

61. Chassang M, Grimaud A, Cucchi JM, Novellas S, Amoretti N, Chevallier P, Bruneton JN. Can low-dose computed tomographic scan of the spine replace conventional radiography? An evaluation based on imaging myelomas, bone metastases, and fractures from osteoporosis. *Clinical imaging* 2007; **31**(4): 225-7.

62. Mahnken AH, Wildberger JE, Gehbauer G, Schmitz-Rode T, Blaum M, Fabry U, Gunther RW. Multidetector CT of the spine in multiple myeloma: comparison with MR imaging and radiography. *AJR American journal of roentgenology* 2002; **178**(6): 1429-36.

63. Hur J, Yoon CS, Ryu YH, Yun MJ, Suh JS. Efficacy of multidetector row computed tomography of the spine in patients with multiple myeloma: comparison with magnetic resonance imaging and fluorodeoxyglucose-positron emission tomography. *Journal of computer assisted tomography* 2007; **31**(3): 342-7.

64. Horger M, Claussen CD, Bross-Bach U, Vonthein R, Trabold T, Heuschmid M, Pfannenberg C. Whole-body low-dose multidetector row-CT in the diagnosis of multiple myeloma: an alternative to conventional radiography. *European journal of radiology* 2005; **54**(2): 289-97.

65. Kyle RA, Durie BG, Rajkumar SV, Landgren O, Blade J, Merlini G, Kroger N, Einsele H, Vesole DH, Dimopoulos M, San Miguel J, Avet-Loiseau H, Hajek R, Chen WM, Anderson KC, Ludwig H, Sonneveld P, Pavlovsky S, Palumbo A, Richardson PG, Barlogie B, Greipp P, Vescio R, Turesson I, Westin J, Boccadoro M, International Myeloma Working G. Monoclonal gammopathy of undetermined significance (MGUS) and smoldering (asymptomatic) multiple myeloma: IMWG consensus perspectives risk factors for progression and guidelines for monitoring and management. *Leukemia* 2010; **24**(6): 1121-7.

66. Bird J, Behrens J, Westin J, Turesson I, Drayson M, Beetham R, D'Sa S, Soutar R, Waage A, Gulbrandsen N, Gregersen H, Low E, Haemato-oncology Task Force of the British Committee for Standards in Haematology UKMF, Nordic Myeloma Study G. UK Myeloma Forum (UKMF) and Nordic Myeloma Study Group (NMSG): guidelines for the investigation of newly detected M-proteins and the management of monoclonal gammopathy of undetermined significance (MGUS). *British journal of haematology* 2009; **147**(1): 22-42.

67. Sigurdardottir EE, Turesson I, Lund SH, Lindqvist EK, Mailankody S, Korde N, Bjorkholm M, Landgren O, Kristinsson SY. The Role of Diagnosis and Clinical Follow-up of Monoclonal Gammopathy of Undetermined Significance on Survival in Multiple Myeloma. *JAMA oncology* 2015; **1**(2): 168-74.

68. Bianchi G, Kyle RA, Colby CL, Larson DR, Kumar S, Katzmann JA, Dispenzieri A, Therneau TM, Cerhan JR, Melton LJ, 3rd, Rajkumar SV. Impact of optimal follow-up of monoclonal gammopathy of undetermined significance on early diagnosis and prevention of myeloma-related complications. *Blood* 2010; **116**(12): 2019-25; quiz 197.

69. Go RS, Gundrum JD, Neuner JM. Determining the clinical significance of monoclonal gammopathy of undetermined significance: a SEER-Medicare population analysis. *Clinical lymphoma, myeloma & leukemia* 2015; **15**(3): 177-86 e4.

70. Mateos MV, Hernandez MT, Giraldo P, de la Rubia J, de Arriba F, Lopez Corral L, Rosinol L, Paiva B, Palomera L, Bargay J, Oriol A, Prosper F, Lopez J, Olavarria E, Quintana N, Garcia JL, Blade J, Lahuerta JJ, San Miguel JF. Lenalidomide plus dexamethasone for high-risk smoldering multiple myeloma. *The New England journal of medicine* 2013; **369**(5): 438-47.

71. Kyle RA, Rajkumar SV. Monoclonal Gammopathy of Undetermined Significance and Multiple Myeloma. *JAMA oncology* 2015; **1**(2): 174-5.

72. Rajkumar SV. Prevention of progression in monoclonal gammopathy of undetermined significance. *Clinical cancer research : an official journal of the American Association for Cancer Research* 2009; **15**(18): 5606-8.

73. Hjorth M, Hellquist L, Holmberg E, Magnusson B, Rodjer S, Westin J. Initial versus deferred melphalan-prednisone therapy for asymptomatic multiple myeloma stage I--a randomized study. Myeloma Group of Western Sweden. *Eur J Haematol* 1993; **50**(2): 95-102.

74. Rajkumar SV, Dispenzieri A, Fonseca R, Lacy MQ, Geyer S, Lust JA, Kyle RA, Greipp PR, Gertz MA, Witzig TE. Thalidomide for previously untreated indolent or smoldering multiple myeloma. *Leukemia* 2001; **15**(8): 1274-6.

75. Barlogie B, van Rhee F, Shaughnessy JD, Jr., Epstein J, Yaccoby S, Pineda-Roman M, Hollmig K, Alsayed Y, Hoering A, Szymonifka J, Anaissie E, Petty N, Kumar NS, Srivastava G, Jenkins B, Crowley J, Zeldis JB. Seven-year median time to progression with thalidomide for smoldering myeloma: partial response identifies subset requiring earlier salvage therapy for symptomatic disease. *Blood* 2008; **112**(8): 3122-5.

76. Korde N, Roschewski M, Zingone A, Kwok M, Manasanch EE, Bhutani M, Tageja N, Kazandjian D, Mailankody S, Wu P, Morrison C, Costello R, Zhang Y, Burton D, Mulquin M, Zuchlinski D, Lamping L, Carpenter A, Wall Y, Carter G, Cunningham SC, Gounden V, Sissung TM, Peer C, Maric I, Calvo KR, Braylan R, Yuan C, Stetler-Stevenson M, Arthur DC, Kong KA, Weng L, Faham M, Lindenberg L, Kurdziel K, Choyke P, Steinberg SM, Figg W, Landgren O. Treatment With Carfilzomib-Lenalidomide-Dexamethasone With Lenalidomide Extension in Patients With Smoldering or Newly Diagnosed Multiple Myeloma. *JAMA oncology* 2015.

77. Golombick T, Diamond TH, Badmaev V, Manoharan A, Ramakrishna R. The potential role of curcumin in patients with monoclonal gammopathy of undefined significance--its effect on paraproteinemia and the urinary N-telopeptide of type I collagen bone turnover marker. *Clinical cancer research : an official journal of the American Association for Cancer Research* 2009; **15**(18): 5917-22.

78. Chang SH, Luo S, O'Brian KK, Thomas TS, Colditz GA, Carlsson NP, Carson KR. Association between metformin use and transformation of monoclonal gammopathy of undetermined significance to multiple myeloma in U.S. veterans with diabetes mellitus: a population-based cohort study. *The Lancet Haematology* 2015; **2**(1): e30-e6.

79. Vu T, Gonsalves W, Kumar S, Dispenzieri A, Lacy MQ, Buadi F, Gertz MA, Rajkumar SV. Characteristics of exceptional responders to lenalidomide-based therapy in multiple myeloma. *Blood cancer journal* 2015; **5**: e363.

80. Shekelle PG. Commentary on N Saquib et al. Does screening for disease save lives in asymptomatic adults? Systematic review of 5 meta-analyses and randomized trials. *International journal of epidemiology* 2015; **44**(1): 277-8.

81. Saquib N, Saquib J, Ioannidis JP. Does screening for disease save lives in asymptomatic adults? Systematic review of meta-analyses and randomized trials. *International journal of epidemiology* 2015; **44**(1): 264-77.

82. Carroll AE. How useful are screening tests? *JAMA : the journal of the American Medical Association* 2015; **313**(13): 1304.

83. Taylor KL, Shelby R, Gelmann E, McGuire C. Quality of life and trial adherence among participants in the prostate, lung, colorectal, and ovarian cancer screening trial. *Journal of the National Cancer Institute* 2004; **96**(14): 1083-94.

84. Kapidzic A, Korfage IJ, van Dam L, van Roon AH, Reijerink JC, Zauber AG, van Ballegooijen M, Kuipers EJ, van Leerdam ME. Quality of life in participants of a CRC screening program. *British journal of cancer* 2012; **107**(8): 1295-301.

85. Taupin D, Chambers SL, Corbett M, Shadbolt B. Colonoscopic screening for colorectal cancer improves quality of life measures: a population-based screening study. *Health and quality of life outcomes* 2006; **4**: 82.

86. Cormier L, Guillemin F, Valeri A, Fournier G, Cussenot O, Mangin P, Litwin MS. Impact of prostate cancer screening on health-related quality of life in at-risk families. *Urology* 2002; **59**(6): 901-6.

87. Wardle FJ, Collins W, Pernet AL, Whitehead MI, Bourne TH, Campbell S. Psychological impact of screening for familial ovarian cancer. *Journal of the National Cancer Institute* 1993; **85**(8): 653-7.

88. Brett J, Bankhead C, Henderson B, Watson E, Austoker J. The psychological impact of mammographic screening. A systematic review. *Psycho-oncology* 2005; **14**(11): 917-38.

89. Sutton S, Saidi G, Bickler G, Hunter J. Does routine screening for breast cancer raise anxiety? Results from a three wave prospective study in England. *Journal of epidemiology and community health* 1995; **49**(4): 413-8.

90. Heijnsdijk EA, Wever EM, Auvinen A, Hugosson J, Ciatto S, Nelen V, Kwiatkowski M, Villers A, Paez A, Moss SM, Zappa M, Tammela TL, Makinen T, Carlsson S, Korfage IJ, Essink-Bot ML, Otto SJ, Draisma G, Bangma CH, Roobol MJ, Schroder FH, de Koning HJ. Quality-of-life effects of prostate-specific antigen screening. *The New England journal of medicine* 2012; **367**(7): 595-605.

91. Wilson JM, Jungner YG. [Principles and practice of mass screening for disease]. *Boletin de la Oficina Sanitaria Panamericana Pan American Sanitary Bureau* 1968; **65**(4): 281-393.

92. Sigurdardottir LG, Jonasson JG, Stefansdottir S, Jonsdottir A, Olafsdottir GH, Olafsdottir EJ, Tryggvadottir L. Data quality at the Icelandic Cancer Registry: comparability, validity, timeliness and completeness. *Acta oncologica* 2012; **51**(7): 880-9.

93. Weiss BM, Minter A, Abadie J, Howard R, Ascencao J, Schechter GP, Kuehl M, Landgren O. Patterns of monoclonal immunoglobulins and serum free light chains are significantly different in black compared to white monoclonal gammopathy of undetermined significance (MGUS) patients. *American journal of hematology* 2011; **86**(6): 475-8.

APPENDIX: LIST OG QUESTIONNAIRES

At the time of informed consent, if given online on www.mgus.is.

Quality of Life Questionnaires

- Patient Health Questionnaire (PHQ-9)
- Generlized Anxiety Disorder (GAD-7)
- Satisfaction with Life Scale (SWLS)
- Happiness Question
- Question of Well Being

At visits to the clinical study center

Background questions (only at first visit)

Physical and Mental Health Questionnaires

- Brief Pain Inventory
- Neuropathy Symptom Score
- Douleur Neuropathique 4 (DN-4)

Quality of Life Questionnaires

- Patient Health Questionnaire (PHQ-9)
- Generalized Anxiety Disorder (GAD-7)
- Satisfaction with Life Scale (SWLS)
- 36-Item Short Form Survey (SF-36)
- Perceived Stress Scale (PSS-10)
- Posttraumatic stress disorder checklist for DMS-5 (PCL-5)
- Happiness Question
- Question of Well Being

Yearly for all participants providing an e-mail address

Background questions (only at first round)

Physical and Mental Health Questionnaires

- Brief Pain Inventory
- Neuropathy Symptom Score
- Douleur Neuropathique 4 (DN-4)

Quality of Life Questionnaires

- Patient Health Questionnaire (PHQ-9)
- Generlazed Anxiety Disorder (GAD-7)
- Satisfaction with Life Scale (SWLS)
- 36-Item Short Form Survey (SF-36)

- Perceived Stress Scale (PSS-10)
- Happiness Question
- Question of Well Being

Nutrition questions – will be sent out to all participants within the next 2 years.
